# Expanding Chemistry of Expanded Helicenes

**DOI:** 10.1002/chem.202502193

**Published:** 2025-09-02

**Authors:** Shinji Toyota

**Affiliations:** ^1^ Department of Chemistry School of Science Institute of Science Tokyo 2–12–1 Ookayama, Meguro‐ku Tokyo 152–8551 Japan

**Keywords:** arenes, chirality, expanded helicenes, helical structures, molecular dynamics

## Abstract

Expanded helicenes are interesting compounds created by modifying the original helicene structure through the incorporation of linearly fused benzene rings, enlarging the helical diameter. Motivated by Tilley et al.’s report of a key expanded helicene structure in 2017, several research groups have synthesized such nonplanar aromatic compounds, aiming to explore their impressive structures, properties, and chiroptical performance. This review highlights recent advances in the expanded helicene chemistry through experimental and theoretical studies. The shape and length of helical structures depend on the number and combination of angularly and linearly fused benzene rings. Helical structures are classified using notations, and specific compounds corresponding to each structural form, namely, hexagonal, triangular, rhombic, or others, are introduced herein. As an extension of the molecular design, examples of nonhexagonal and heteroaromatic ring‐embedded expanded helicenes are presented. Specifically, this review focuses on how the diameters, lengths, and turn numbers of helical structures depend on dynamic processes involving helical inversion and chiroptical properties (circular dichroism (CD) and circularly polarized luminescence (CPL)). The characteristics and perspectives of this molecular design are also discussed.

## Graphical abstract

This review presents recent advances in the chemistry of expanded helicenes, defined as helicenes containing linearly fused benzene rings, as novel aromatic compounds. This review begins with a summary of their structural classification, followed by the synthesis, structures, properties, and chiroptical performance of expanded helicenes of various shapes, such as hexagons, triangles, and rhombuses.

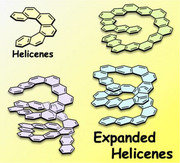

## 1 Introduction

**Figure 1:**
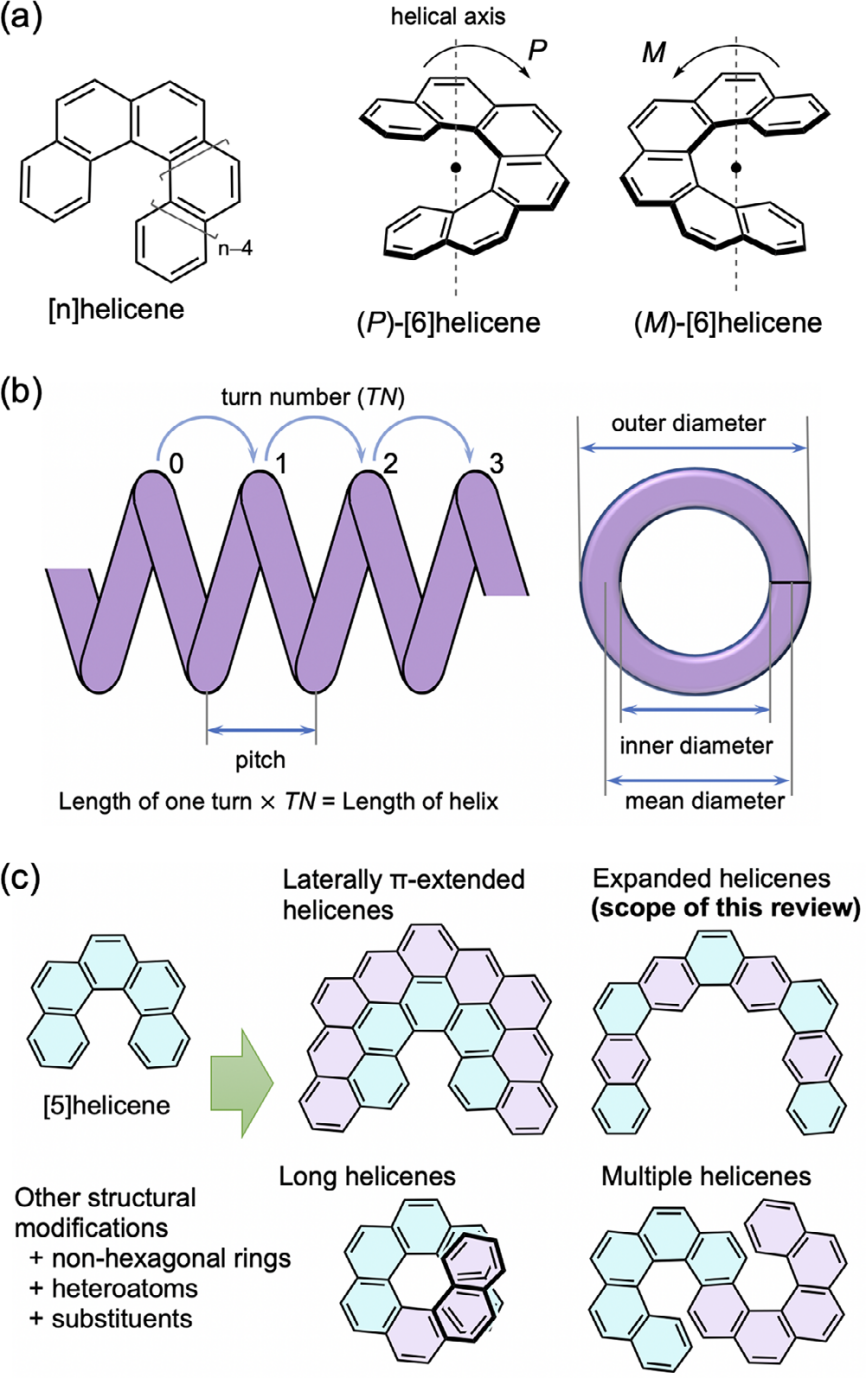
a) Structures of [n]helicene and enantiomers of [6]helicene. b) Key dimensional parameters of a schematic helical object. c) Structural modifications of [n]helicenes starting from [5]helicene as a typical example. Original and added benzene rings are highlighted in light blue and light purple, respectively.

Helicenes are fascinating motifs for constructing nonplanar helically shaped structures by fusing aromatic units in an angular fashion.^[^ ^1^, ^2^, ^3^, ^4^, ^5^, ^6^ ^]^ According to the IUPAC Recommendation, helicenes are defined as “*ortho*‐fused polycyclic aromatic or heteroaromatic compounds in which all rings (minimum five) are angularly arranged so as to give helically shaped molecules.” Here “*ortho*‐fusion” refers to polycyclic compounds in which two rings share two atoms.^[^ ^7^, ^8^ ^]^ The most popular example of [n]helicenes comprising n angularly fused benzene rings, the most popular example is [6]helicene (Figure 1a). The steric hindrance between the terminal benzene rings deforms the aromatic framework, resulting in a nonplanar helical structure that is chiral. Such a compound has a helical chirality, and its stereochemistry is designated as *M* (minus) or *P* (plus) depending on the direction, left‐ or right‐handed, respectively, as one moves along the helical axis from front to back.^[^ ^9^ ^]^ The first synthesis of [6]helicene was reported by Newman et al. in 1955,^[^ ^10^, ^11^ ^]^ and the resolved enantiomers showed a very large magnitude of specific rotation [α]_D_ = ca. ±3600. The absolute stereochemistry of the enantiomers was first established in 1971 using X‐ray diffraction data by the Bijvoet method.^[^ ^12^, ^13^ ^]^ Because of their interesting structures and properties, [6]helicene and its analogs have been studied by numerous researchers in organic and aromatic chemistry, as well as related fields such as chiral, supramolecular, and functional material chemistry.^[^ ^14^, ^15^, ^16^, ^17^, ^18^, ^19^ ^]^ Helicenes continue to be an intriguing topic in modern chemistry.

Aiming to create new helicenes with novel structures and properties, researchers have developed various approaches for the design of helical structures based on the original helicene over a long time. These approaches can be broadly classified as modification of the helical framework, introduction of substituents, or a combination of helical moieties. In the first approach, the helical geometry is influenced by the molecular structures. Similar to a mechanical helical spring, the geometry is characterized by three parameters: pitch (the height of one helical turn), diameter, and length, as illustrated in Figure 1b.^[^ ^20^, ^21^, ^22^, ^23^, ^24^ ^]^ These parameters automatically determine the turn number (*TN*). Additionally, the force constant for the compression–expansion motion is a key parameter for evaluating dynamic behavior in analogy to a mechanical spring.^[^ ^22^, ^23^, ^24^, ^25^, ^26^ ^]^

There are several ways to enlarge the helical structures (Figure 1c). First, the helical chain can be lengthened by adding extra benzene rings to increase the helical turn while retaining the helical diameter. Long [n]helicenes are challenging synthetic targets for creating highly coiled helical molecules.^[^ ^27^, ^28^ ^]^ The longest analog synthesized thus far is [16]helicene, which has a *TN* of ca. 2.5.^[^ ^29^ ^]^ The second way involves fusing extra benzene rings to the outer rim of the helical structure. This modification increases the outer diameter without altering the inner diameter. Such compounds, called “laterally π‐extended helicenes” (hereinafter, “extended helicenes”),^[^ ^30^ ^]^ which are interesting as models of twisted nanographenes with wide π‐surfaces.^[^ ^31^, ^32^, ^33^, ^34^, ^35^, ^36^, ^37^, ^38^, ^39^, ^40^ ^]^ In these extended helicenes, the width of a helical ribbon exceeds that of one benzene ring; that is, the difference between the inner and outer diameters increases. The third way involves adding linearly fused benzene rings to the helicene structure to increase the outer and inner diameters of the original helicene structure. Such molecules consisting of all *ortho*‐fused (also called *cata*‐fused)^[^ ^41^, ^42^ ^]^ benzene rings are called “expanded helicenes.”^[^ ^30^, ^43^ ^]^ Unlike the above‐mentioned modifications of a single helical structure, assembling more than one helical moiety into a molecule produces “multiple‐helicenes,” as exemplified by S‐shaped double helicenes.^[^ ^44^, ^45^, ^46^, ^47^, ^48^, ^49^, ^50^ ^]^ Depending on the number and orientation of helical sites, these compounds exhibit remarkable stereochemical and spectroscopic behavior, particularly chiroptical properties such as circular dichroism (CD) and circularly polarized luminescence (CPL).^[^ ^30^, ^51^, ^52^, ^53^ ^]^ In addition, we can incorporate nonhexagonal rings, such as pentagons and heptagons, to form irregular helicenes, sometimes called “nonbenzenoid helicenes”.^[^ ^54^, ^55^, ^56^, ^57^, ^58^, ^59^, ^60^ ^]^ We can also incorporate heteroatoms, such as N, S, B, and O atoms, into helicene structures. Such helicenes are called “heterohelicenes” to distinguish them from “cabohelicenes”, which consist only of carbon and hydrogen atoms.^[^ ^61^, ^62^, ^63^, ^64^, ^65^, ^66^, ^67^ ^]^ Combinations of these modifications produce an infinite number of helicene structures. Thus, the molecular design of helicene‐based compounds is highly versatile.

**Figure 2:**
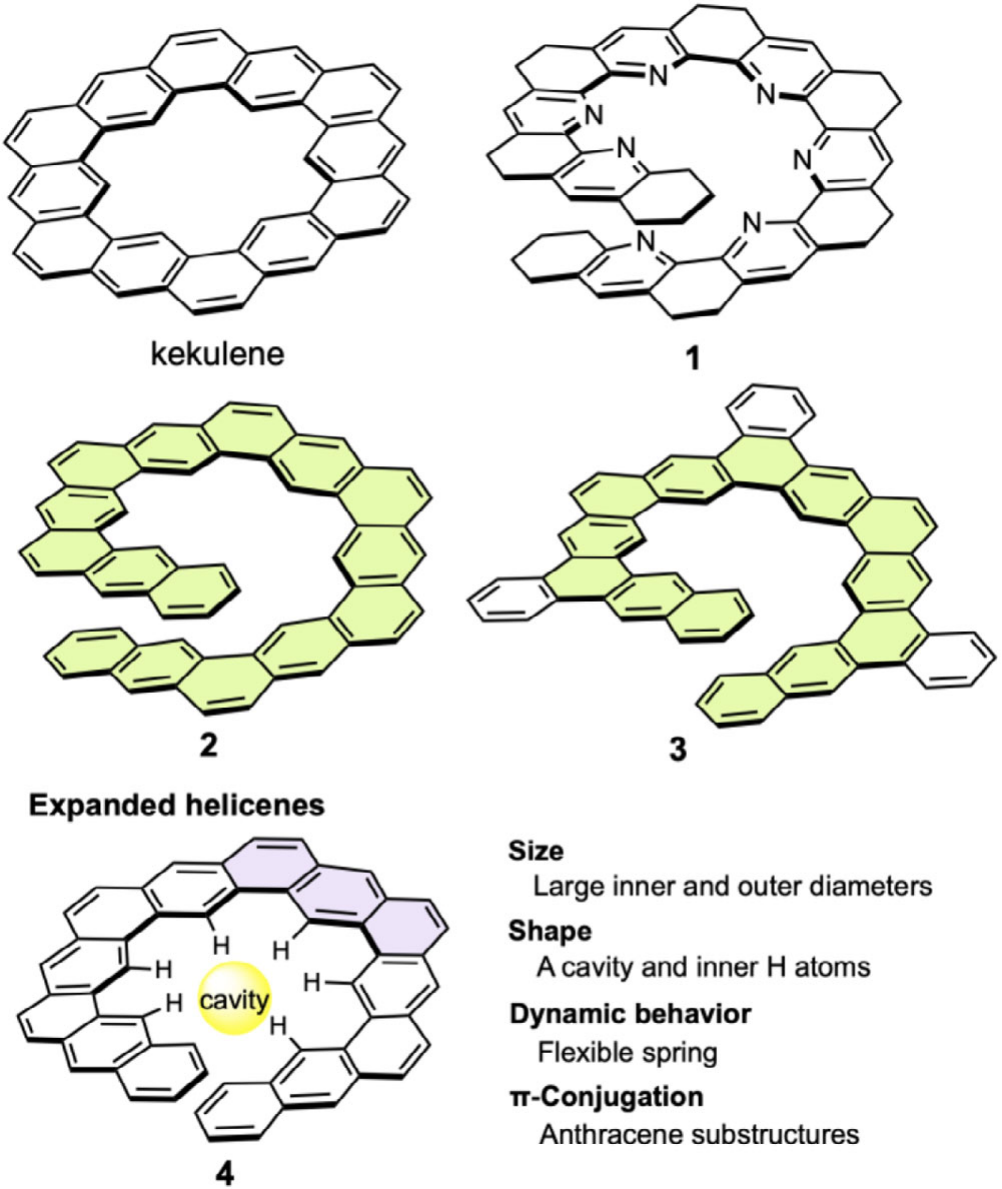
Fundamental and related structures of expanded helicenes and their features. Substituents are omitted for **1** and **3**. Benzene rings in the expanded helical chains are highlighted in light green in **2** and **3**. An anthracene substructure is highlighted in light purple in **4**.

In 1991, Bell et al. first used the term “expanded” to describe the structure of *N‐*containing partially saturated heterohelicene **1** (Figure 2).^[^ ^68^ ^]^ In that article, they proposed a conceptual model of expanded carbohelicene structure **2**, which is analogous to kekulene, a polycyclic aromatic hydrocarbon (PAH) consisting of 12 circularly fused benzene rings. Later, Tilley et al. synthesized fully aromatized expanded helicene **3** with 13 benzene rings, not counting the additional fused benzene rings.^[^ ^30^ ^]^ This was the first key compound in the chemistry of expanded helicenes. They defined “expanded helicenes” as helicenes composed of alternating linearly and angularly fused rings. Today, this term is broadly used to describe helicenes with any number or combination of linearly fused rings.

In general, expanded helicenes such as **4** possess the characteristics shown in Figure 2. The incorporation of linearly fused rings increases their helical size in the radial direction. Consequently, expanded helicenes typically have a cavity surrounded by H atoms in the inner region, in contrast to the original helicenes without inner H atoms. When the cavity is sufficiently large, it can accommodate foreign chemical species and construct separate spaces or channels in the solid state. From a dynamic perspective, expanded helicenes are more flexible than extended helicenes with similar outer diameters.^[^ ^23^, ^43^ ^]^ Therefore, as the spring‐like expansion/contraction motion of expanded helicenes occurs rapidly, the resolution of enantiomers should require a long helical chain or additional steric hindrance to prevent racemization via helical inversion. Expanded helicenes have a wide π‐electron surface with multiple anthracene or longer acene substructures; therefore, their π‐conjugation is extended compared to that of the original helicenes. This structural feature significantly influences the molecular orbital levels, affecting the photophysical and electrochemical properties.

Following the report on key expanded helicenes by Tilley et al. in 2017,^[^ ^30^ ^]^ several expanded helicenes have been synthesized by his group and other groups, underpinned by remarkable advances in synthetic, instrumental, and computational methods. In this regard, it is timely to review expanded helicenes that have been studied so far, and compare their structures, as well as the electronic, photophysical, and chiroptical properties, with those of other helicenes or nanographenes in order to clarify the advantages of this molecular design. In the next section, known expanded helicenes are classified on the basis of their structural patterns to facilitate understanding of their complicated structures. In the sections that follow, examples of expanded helicenes consisting of hexagonal benzene units are discussed by structural shape: hexagonal, triangular, rhombic, and others. Representative examples of expanded helicenes embedded with nonhexagonal and heteroaromatic rings are also presented. The last section summarizes the current state of expanded helicene chemistry and provides an outlook for the developments of this molecular design.

## 2 Classification of Expanded Helicenes

**Figure 3:**
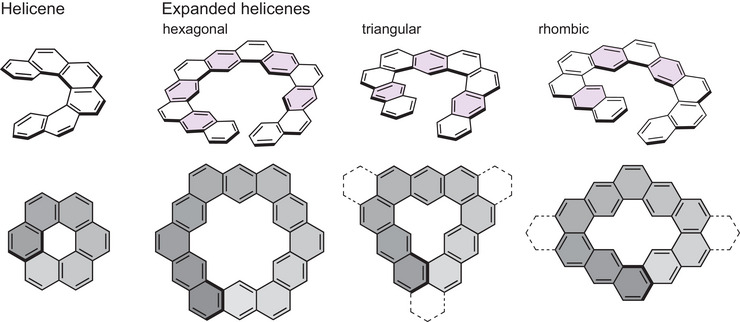
Structures of original helicene and typical expanded helicenes for one helical turn, and their perspective views along the helical axis. Linear fused benzene rings are highlighted in light purple in the top structures. In some perspective views, additional hexagons are drawn by broken lines to apply the revised Porsev's method.

It is essential to classify the structures of expanded helicenes in a general manner to distinguish the geometric differences, because the IUPAC nomenclature of such compounds is so complicated. The geometric analysis and symbols of the original helicene and the typical expanded helicenes shown in Figure 3 are listed in Table 1.

**Table 1:** Classifications of helicene and expanded helicenes, their geometric representations, and related information.

	Original helicene	Hexagonal expanded helicene	Triangular expanded helicene	Rhombic expanded helicene
shape	hexagon	hexagon	triangle	rhombus
*N* ^[^ ^a^ ^]^	6	12	9	10
*n* ^[^ ^b^ ^]^	up to 16	13, 15, 19, 23	9, 12, 13, 15, 21	11, 19, 27, 35
AL notation ^[^ ^c^ ^]^	AAAA	LALALALALA	LAALAAL	LAALALAA
Balaban's code	[1111]	[0101010101]	[0110110]	[01101011]
Hirose and Matsuda's method	[6,6^2^,6^2^,6^2^,6^2^,6]	[6,6^1^,6^2^,6^1^,6^2^,6^1^,6^2^,6^1^,6^2^, 6^1^,6^2^,6]	[6,6^1^,6^2^,6^2^,6^1^,6^2^,6^2^,6^1^,6]	[6,6^1^,6^2^,6^2^,6^1^,6^2^,6^1^,6^2^,6^2^,6]
Simplified Hirose and Matsuda's method	[6,(6^2^)_4_,6]	[6,(6^1^,6^2^)_5_,6]	[6,(6^1^,6^2^,6^2^)_2_,6^1^,6]	[6,6^1^,6^2^,(6^2^,6^1^)_2_,6^2^,6^2^,6]
Porsev's method	[1.1]	[2.1]	–	–
revised Porsev's method	$[1_{zz}^{h}.1_{zz}^{h}$]	$[2_{zz}^{h}.1_{zz}^{h}$]	$[1_{zz}^{t3}.2_{zz}^{t3}$]^[^ ^d^ ^]^	$[1_{zz}^{r2}.2_{zz}^{r2}$]^[^ ^d^ ^]^
2D analog ^[^ ^e^ ^]^	coronene	Kekulene	coronoid	coronoid
substructure^[^ ^f^ ^]^	–	[3]helicene	[4]helicene	[3]helicene [4]helicene

^[a]^ Number of benzene rings required for one helical turn.

^[b]^ Number of benzene rings in known compounds.

^[c]^ Angular (A) and linear (L) notations.

^[d]^ Additional hexagons at corners shown by broken lines are conveniently considered (see Figure 3).

^[e]^ Structures of two‐dimensional (2D) perspective view along the helical axis.

^[f]^ Substructures found along the inner rim.

**Figure 4:**
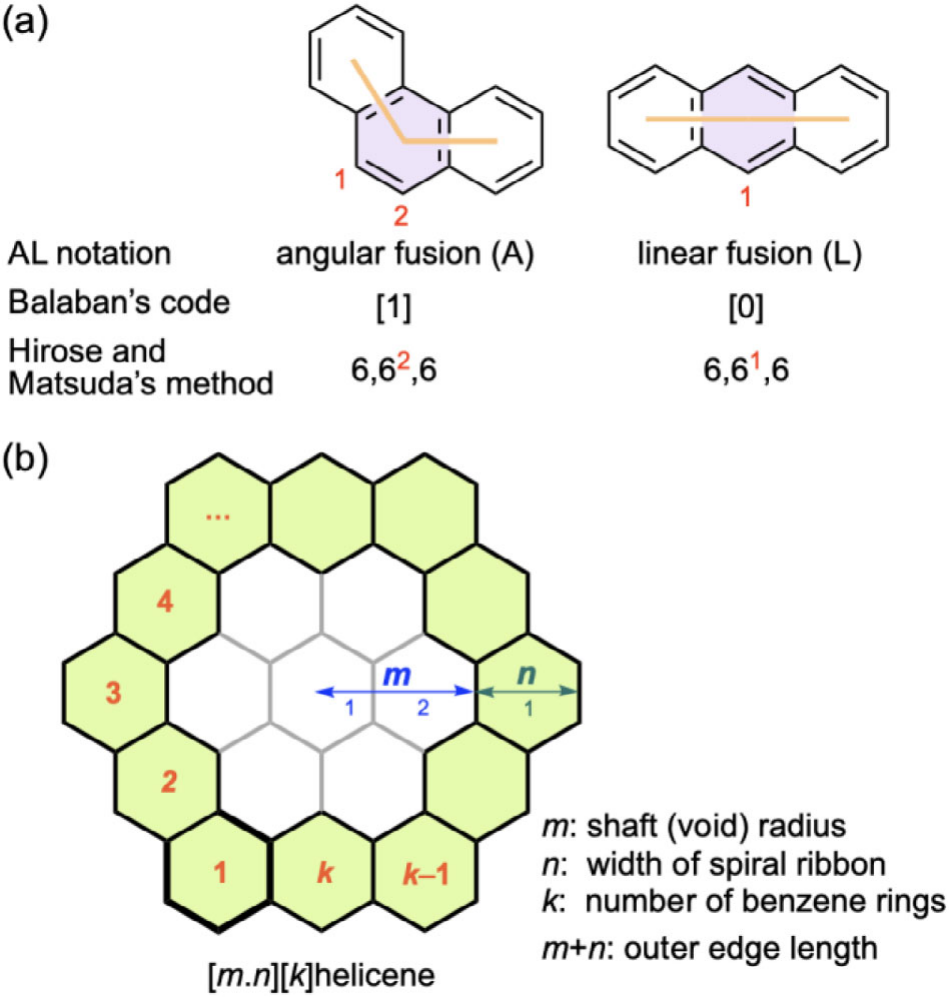
a) Notations for angular and linear fusions of three consecutive benzene rings. b) Key parameters to represent expanded helicene structures using Porsev's method.

Similar to the original helicene, the number of benzene rings in the helical chain is indicated in brackets, e.g. [6]helicene. However, this convention does not provide information on the direction of the fused rings for expanded helicenes. In *ortho*‐fused polycyclic aromatic compounds, three consecutive benzene rings are fused angularly at 120° (A) or linearly at 180° (L) (Figure 4a).^[^ ^69^, ^70^, ^71^ ^]^ The fused pattern of [6]helicene is represented by “AAAA”, namely, all A's, for its four nonterminal benzene rings. A one‐turn helical unit of Tilley's expanded helicene with a hexagonal shape is represented as “LALALALALA”, namely, alternating L's and A's. Balaban et al. proposed a similar code system to define the structures of *cata*‐fused aromatic compounds.^[^ ^72^, ^73^ ^]^ The digit 0 indicates linear fusion, and digits 1 and 2 indicate angular fusion depending on the direction (only digit 1 appears for helicenes). For example, [6]helicene is represented as [1111]. Digits 0 and 1 correspond to L and A, respectively, in the AL notation. In both systems, the number of letters or digits is equal to the number of benzene rings minus two because the terminal benzene rings are not considered.

Hirose and Matsuda proposed a nomenclature for expanded helicenes^[^ ^43^ ^]^ based on the nomenclature for the corannulene system proposed by Agranat et al.^[^ ^74^ ^]^ A hexagonal helicene consisting of 12 benzene rings, shown in Figure 3, is named helicene[6,6^1^,6^2^,6^1^,6^2^,6^1^,6^2^,6^1^,6^2^,6^1^,6^2^,6], where the number 6 indicates a six‐membered ring and superscripts 1 (linear) and 2 (angular) indicate the number of outer carbon atoms not shared by the adjacent rings (Figure 4a). This number is so long that it can be shortened to helicene[6,(6^1^,6^2^)_5_,6], where repeating terms are simplified. The number of algebraic expressions separated by commas is equal to the number of aromatic rings in the helical framework. This system is applicable to helicenes with nonhexagonal rings.

Porsev et al. proposed a nomenclature system for characterizing various helical structures.^[^ ^24^ ^]^ They defined the shaft radius (*m*), the width of the spiral ribbon (*n*), and the number of benzene rings in the helical chain (*k*) (Figure 4b). The shaft radius is determined by counting the number of hexagons from the central hexagon to the inner edge. Such expanded helicenes are represented as [*m*.*n*][*k*]helicene: the original helicene and the hexagonal expanded helicene with one helical turn are expressed as [1.1][6]helicene and [2.1][12]helicene, respectively. By definition, *n *= 1 for expanded helicenes, whereas *n *≥ 2 for laterally extended helicenes. The length of the outer edge is equal to *m *+ *n*, which indicates the radial size of the enlarged helicenes, regardless of the size of the inner cavity. Specifically, the distance from the center to one of the corners is 2 + 1 = 3 for [2.1][12]helicene. In order to apply the nomenclature to various helical shapes, they proposed a revised method based on the nomenclature of graphene nanoflakes.^[^ ^22^, ^75^ ^]^ For example, the hexagonal expanded helicene is expressed as $[2_{zz}^{h}.1_{zz}^{h}$]helicene, where the first and second sets of symbols, separated by a period, represent the flake shapes of the inner cavity and the aromatic ribbon moiety, respectively. These symbols indicate the dimensions and shapes (*h*: hexagonal, *t*: trigonal, or *r*: rhombus) of the graphene nanoflakes, the position of the central point (6: hexagon center, 3: corner, or 2: side midpoint), and the edge morphology (*zz*: zigzag or *ac*: armchair). For example, the superscript “*t*3” in the nomenclature of the triangular expanded helicene indicates that its structural center is located at the intersection of three hexagons. The complete symbols are shown in Table 1. For convenience, some benzene rings have been removed from the ribbon moiety. All of the helicenes in Table 1 have a zigzag edge (subscript *zz*) along their outer and inner rims.

Table 1 also includes additional information. The perspective views along the helical axis are related to two‐dimensional (2D) planar aromatic analogs by enclosing the terminal moieties. The planar analog of [6]helicene is coronene, and those of the expanded helicenes are coronoids, which are coronene‐like PAHs with a hollow larger than one hexagon.^[^ ^76^, ^77^ ^]^ For the hexagonal expanded helicene, the planar analog is kekulene, a typical coronoid, with seven missing hexagons. The shape of the inner angular rims of the expanded helicene corresponds to any of the [n]helicene substructures. Hexagonal and triangular expanded helicenes have [3]helicene and [4]helicene substructures, respectively, whereas rhombic expanded helicenes have alternating [3]helicene and [4]helicene substructures. In the following sections, we will primarily refer to expanded helicenes by their shapes for intuitive understanding. If necessary, other nomenclature will be used to distinguish similar structures.

## 3 Various Expanded Helicenes

### 3.1 Hexagonal Expanded Helicenes

**Figure 5:**
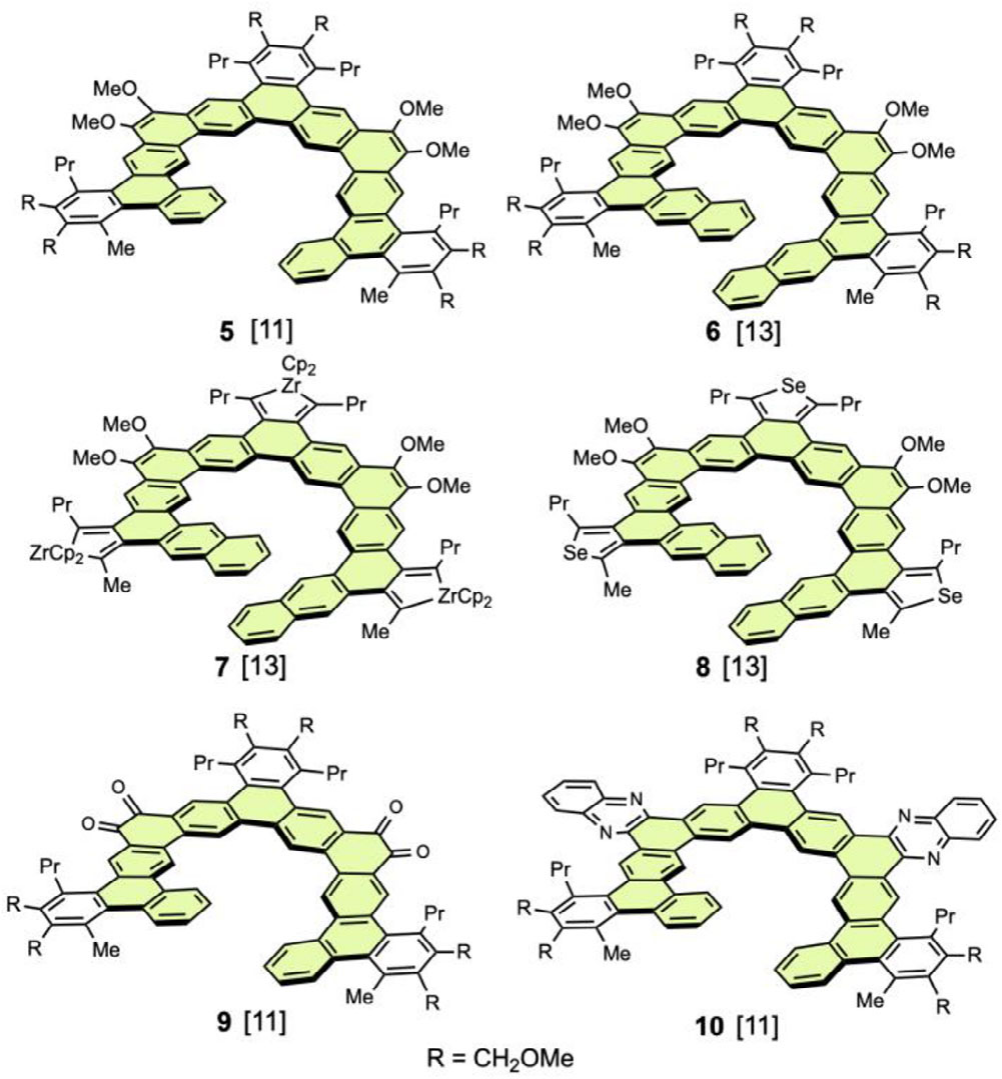
Hexagonal expanded [2.1][n]helicenes. Hereafter, the number of benzene (aromatic) rings along the helical chain is shown in brackets. Main helical ribbons are highlighted in light green. Cp: cyclopentadienyl. Pr: propyl.

In 2017, Tilley et al. synthesized hexagonal helicenes [2.1][11]helicene **5** and [2.1][13]helicene **6** by three‐fold Ir‐catalyzed [2 + 2 + 2]cycloaddition of diyne moieties with an external alkyne (Figure 5).^[^ ^30^ ^]^ X‐ray analysis of [13]helicene **6** revealed a helical structure with a *TN* greater than one and the formation of a homochiral π‐stacked dimer (*MM* or *PP* pair) via long range π‐stacking in the crystal. This helicene was in equilibrium between the monomer and the dimer in CDCl_3_, and the two forms were observed separately at high concentrations using ^1^H NMR spectroscopy. This phenomenon was not observed for the corresponding short analog [11]helicene **5**. Zirconocene and selenophene annulated [2.1][13]helicene derivatives **7** and **8** were also synthesized from the common alkyne precursor. These compounds have low inversion barriers as suggested by the ^1^H NMR spectra at room temperature. The inversion barrier of **7** was estimated to be 45 kJ mol^−1^ at 211 K by variable‐temperature (VT) ^1^H NMR measurement. This barrier is much lower than that of the parent [7]helicene (174 kJ mol^−1^ at 300 K).^[^ ^78^, ^79^, ^80^ ^]^ Therefore, the expanded helicene framework is much more flexible than the original helicene framework. The electronic structures and second‐order nonlinear optical properties of model compounds of expanded helicenes **6** and **8** and their derivatives were studied theoretically for the design of functional materials.^[^ ^81^ ^]^ The structure of **5** was functionalized to quinone **9** and quinoxaline **10** to tune the electronic properties.^[^ ^82^ ^]^ The solid‐state structures of these compounds were analyzed by microcrystal electron diffraction (microED) measurements to reveal their packing behavior and the presence of nanoscale cavities.

**Figure 6:**
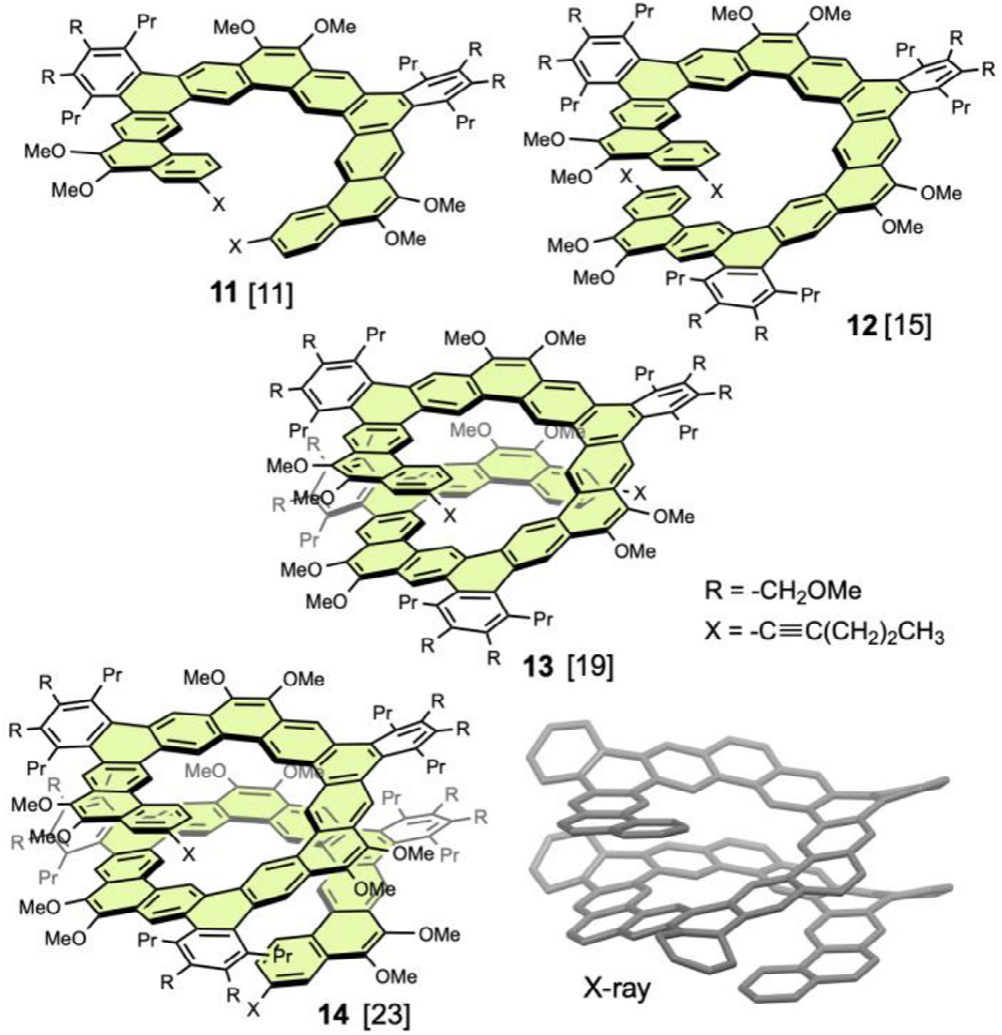
Long hexagonal expanded [2.1][n]helicenes and X‐ray structure of **14** (substituents are omitted).

Then, Tilley et al. synthesized a series of long [*n*]helicenes **11**–**14** (n = 11, 15, 19, and 23, respectively) with terminal 1‐pentynyl groups via an iterative cycloaddition strategy (Figure 6).^[^ ^83^ ^]^ They adopted compound **11** as a synthon for the synthesis of a chiral figure‐eight dimer via alkyne metathesis and arylene‐bridged helicenes via cycloaddition.^[^ ^84^ ^]^ X‐ray analysis revealed that the longest analog **14** has a helical structure with a *TN* close to 2 and the presence of intermolecular π···π interactions at 3.6–4.0 Å (cf. the sum of the van der Waals (vdW) radii of C(sp^2^) atoms, 1.70 + 1.70 = 3.40 Å). The enantiomers of **14** were successfully resolved by chiral HPLC and exhibited characteristic Cotton effects with a large dissymmetry factor of |*g*_abs_| = 0.056, one of the parameters for evaluating the CD response.^[^ ^51^, ^52^ ^]^ The barrier to enantiomerization of **14** was determined by kinetic CD measurements to be 122 kJ mol^−1^. Note that *enantiomerization* is the interconversion of enantiomers as a reversible microscopic process, which differs from *racemization* as an irreversible macroscopic process.^[^ ^85^, ^86^ ^]^ The barriers to enantiomerization increase as the length of the hexagonal helical chains increases, in the order of **11** (<50 kJ mol^−1^), **12** (67 kJ mol^−1^), **13** (96 kJ mol^−1^), and **14**.

**Figure 7:**
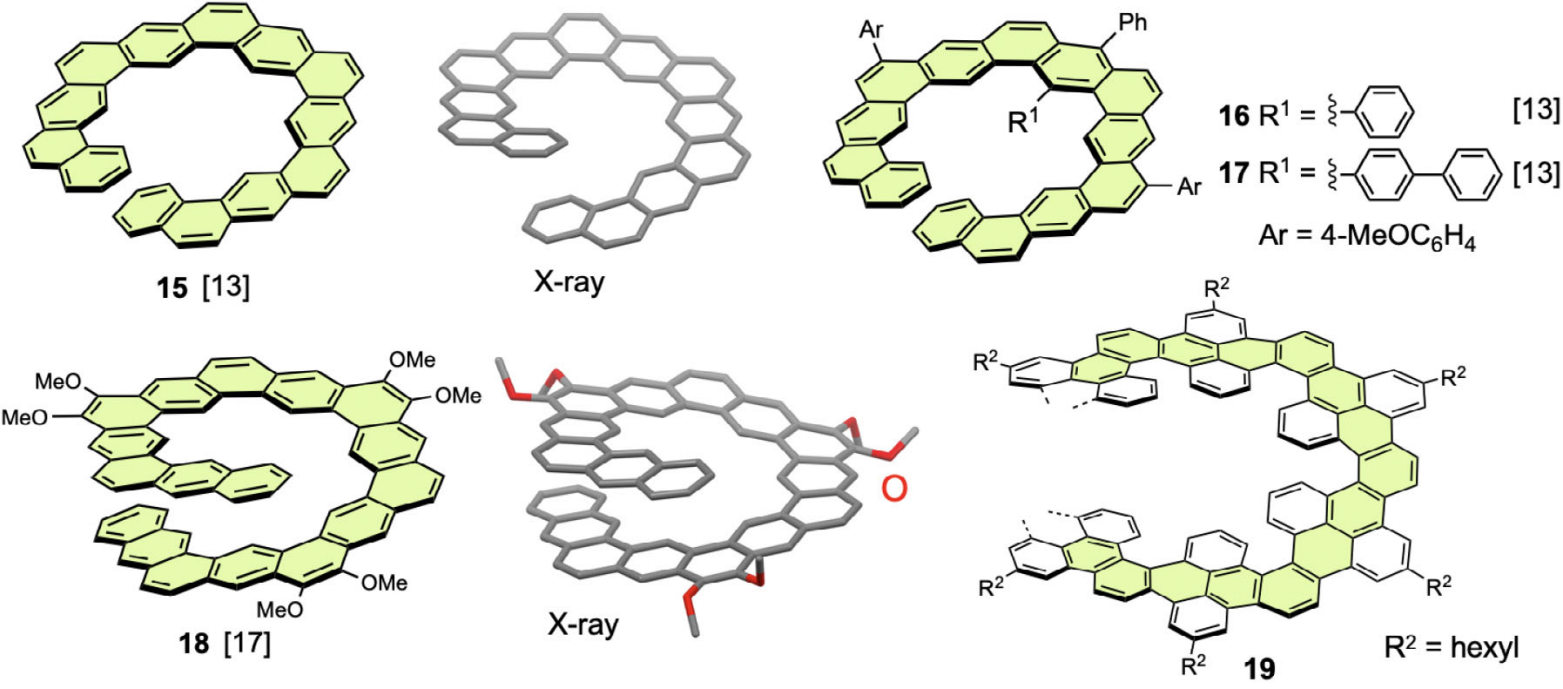
Long hexagonal expanded [2.1][n]helicenes and [3.1][n]helicene and X‐ray structures of **15** and **18**.

In 2018, Hirose and Matsuda et al. reported the synthesis of substituent‐free [2.1][13]helicene **15**, a helical analog of kekulene (Figure 7).^[^ ^43^ ^]^ This expanded helicene was synthesized by six‐fold ring‐closing olefin metathesis in a high yield. According to X‐ray analysis, the molecule has a helical structure, where the mean helical diameter is ca. 10.2 Å (the distance between the benzene centroids) and the angle between the two terminal benzene rings is 18.2°. Structural data and nucleus‐independent chemical shift (NICS) analysis indicated that the aromaticity of the angularly fused benzene rings was less significant than that of the terminal and linearly fused benzene rings. This structural feature is common to other expanded helicenes and other PAHs.^[^ ^87^, ^88^ ^]^ The barrier to enantiomerization of **15** was calculated to be 54 kJ mol^−1^ at the B3LYP/6–311G(2d,p) level. The flexibility of this helical framework as a molecular spring was evaluated by calculation. Assuming Hooke's law, the force constant for the expansion motion of **15** was estimated to be 0.33 N m^−1^. This force constant is much smaller than that of [7]helicene (3.07 N m^−1^), indicating that this expanding helicene acts as a soft molecular spring. Meta and Alcarazo et al. synthesized similar [13]helicene derivatives **16** and **17** with a substituent in the inner fjord region.^[^ ^89^ ^]^ The substituents at the crowded position significantly influenced the helical structure and inversion dynamics. The X‐ray structure of **17** with a linear 4‐phenylphenyl group showed that the helical structure was significantly extended along the helical axis compared to that of **15** to avoid steric hindrance. The mechanism of enantiomerization via helical inversion was analyzed by density functional theory (DFT) calculations using the nudged elastic band (NEB) method.^[^ ^90^ ^]^ The calculated barriers were 59 kJ mol^−1^ for **16** (R^1^ = Ph) and 83 kJ mol^−1^ for **17** (R^1^ = 4‐PhC_6_H_4_), which were higher than that of **15** (54 kJ mol^−1^). Thus, the barrier was enhanced by the long biphenyl substituent.

Long [2.1][15]helicene **18** with methoxy groups was synthesized by Ito and Itami et al. by four‐fold intramolecular Yamamoto coupling of bromoarene precursors (Figure 7).^[^ ^91^ ^]^ The X‐ray structure showed a helical structure with intramolecular π···π stacking between the benzene rings at an interlayer distance of ca. 3.5 Å. Because the *TN* of **18** is ca. 1.3, the interacting π‐surface spreads over five benzene rings at the both terminals, as demonstrated by the noncovalent interaction (NCI) plot.^[^ ^92^ ^]^ DFT calculations suggested that the barrier to enantiomerization of substituent‐free [17]helicene was 117 kJ mol^−1^, which was higher than the barriers of [13]helicene (68 kJ mol^−1^) and [15]helicene (97 kJ mol^−1^) analogs. The enantiomers of **18**, which were resolved by chiral HPLC, racemized at room temperature. However, enantioenriched samples showed CD and CPL activity. The absolute stereochemistry was determined by the time‐dependent (TD) DFT calculation, where the *P* form exhibited an intense positive Cotton effect at 412 nm in the CD spectrum. The barrier to enantiomerization was determined to be 95 kJ mol^−1^ (half‐life 94 min at 298 K) by monitoring the CD intensity. Generally, barriers of 100 kJ mol^−1^ or higher are required for resolution at room temperature without significant racemization.^[^ ^93^ ^]^ Morin et al. synthesized helicene‐like graphene nanoribbon **19** with a [3.1][n]helicene substructure by photochemical cyclization from a polyphenylene precursor.^[^ ^94^ ^]^ This nanoribbon is highly emissive in solution and in the solid state.

**Figure 8:**
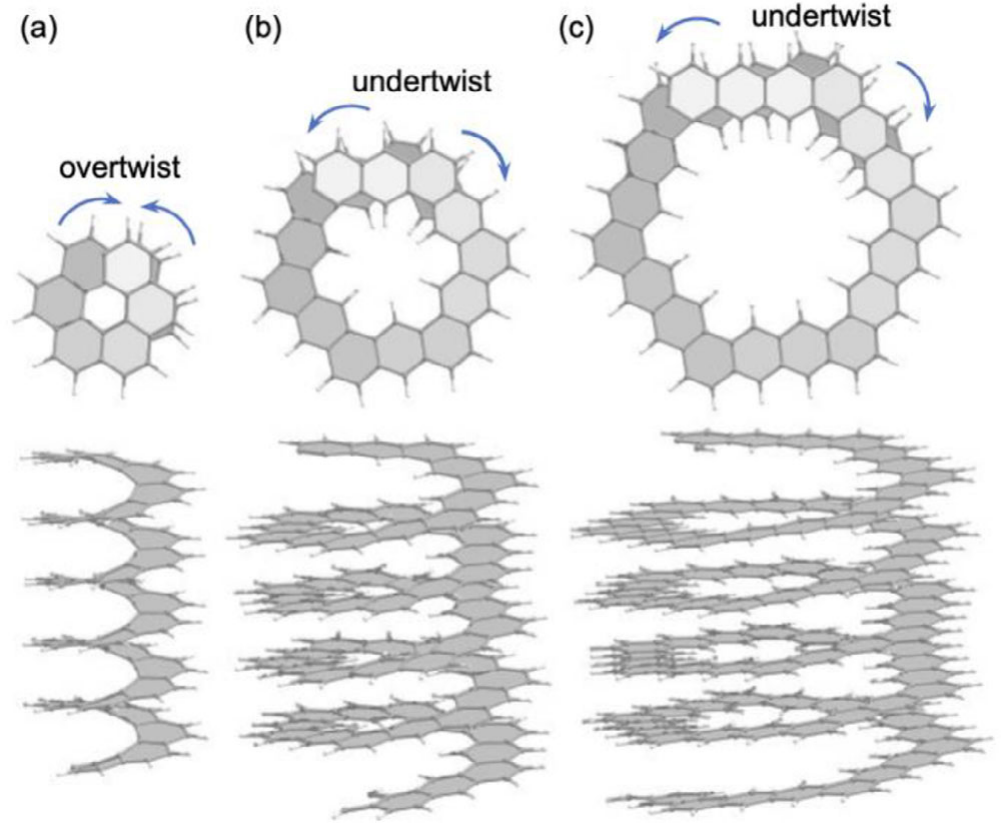
Two views of helical periodicity of global minimum structures of a) [1.1][n]helicene, b) [2.1][n]helicene, and c) [3.1][n]helicene. Perspective views along the helical axes (top) show only one and extra helical turn. Copyright 2022, Elsevier. (ref. 23)

Hexagonal expanded helicenes are an attractive motif in computational chemistry. Lukmanov et al. calculated the thermodynamic and polarizability parameters of a series of [n]helicenes (n = 11–14) and related compounds using the DFT method.^[^ ^95^ ^]^ The calculated data showed that expanded [2.1][n]helicenes were much more stable than the corresponding [1.1][n]helicenes. Additionally, the expanded helicenes and the corresponding circulenes (e.g., [2.1][12]helicene and kekulene) have similar mean polarizability values. Porsev et al. calculated the structures and electronic and magnetic properties of various expanded and extended helicenes as analogs of mechanical springs.^[^ ^22^, ^23^, ^24^ ^]^ In particular, the helical periodicity of hexagonal helicenes was analyzed by DFT calculations (Figure 8).^[^ ^23^ ^]^ Structural optimization gave multiple energy‐minimum structures for [2.1][n] and [3.1][n]helicenes, and the helical structures were undertwisted in the global minimum structures to reduce the *TN* value. In contrast, [1.1][n]helicene had only one energy‐minimum structure, in which the helical structure was slightly overtwisted to increase the *TN* value. Young's modulus values were calculated to evaluate the dependence of axial mechanical properties on torsional deformation. The calculated values were 59.7, 47.1, and 41.2 GPa for the [1.1][n], [2.1][n], and [3.3][n]helicenes, respectively. This trend means that torsional deformation is easy for large expanded helicenes.

### 3.2 Triangular Expanded Helicenes

**Figure 9:**
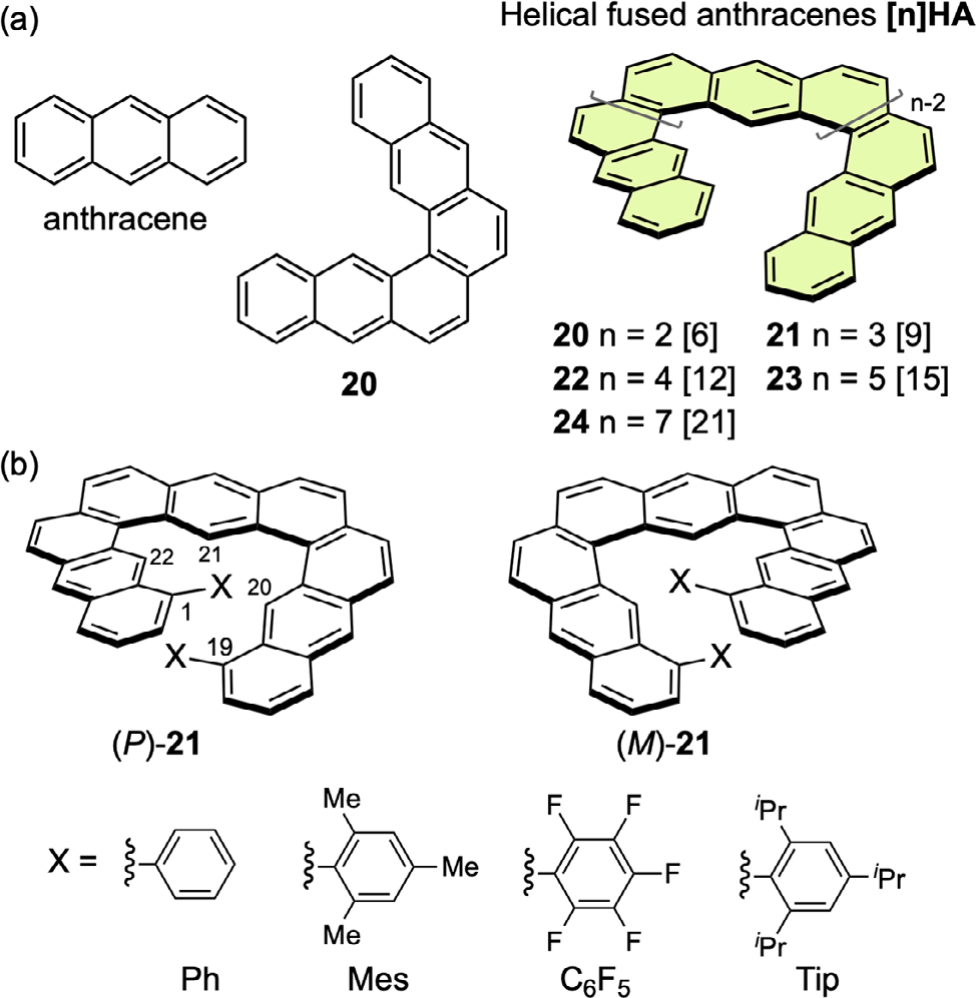
a) Structures of helical fused anthracenes **[n]HA** and b) enantiomers of **21**.

A typical triangular expanded helicene is **[n]HA**, in which n anthracene units are fused at 60° angle in the same direction (Figure 9a). This structure contains n linearly fused benzene rings that are alternately incorporated into [2n]helicene to form a helical structure with 3n benzene rings, and each inner corner has a cove ([4]helicene) structure. The fundamental compound is dianthra[1,2‐*a*:2′,1′‐*j*]anthracene (**21**, n = 3) with three fused anthracene units. This compound was synthesized by PtCl_2_‐catalyzed alkyne cycloisomerization by Toyota et al. in 2020.^[^ ^96^ ^]^ To avoid steric hindrance between the terminal benzene moieties, the molecule adopts a nonplanar helical structure. The calculated barrier to helical inversion of **21** was so low (ca. 25 kJ mol^−1^) that enantiomerization should occur rapidly even at low temperatures.

To retard the helical inversion, derivatives of **21** with various substituents at the sterically crowded 1,19‐positions **21(X)** [Ph, 2,4,6‐trimethylphenyl (Mes), 2,4,6‐triisopropylphenyl (Tip), and C_6_F_5_] were synthesized in a similar manner (Figure 9b).^[^ ^96^, ^97^ ^]^ The enantiomers of these compounds were successfully resolved by chiral HPLC, except for the Mes derivative. For example, enantiopure samples of **21(Ph)** showed high activity in the CD spectra (|Δ*ε*| = 1380 L mol^−1^ cm^−1^, |*g*_abs_| = 0.024 at 352 nm) and CPL spectra (|*g*_lum_| = 0.013). These dissymmetry factors are large for simple organic molecules (typically on the order of 10^−3^–10^−5^).^[^ ^44^, ^51^, ^52^ ^]^ The *g* values are expressed as a function of the transition electronic dipole moment ***μ***, the transition electronic dipole moment ***m***, and the angle *θ* between the two moments: *g* = 4| ***μ*** || ***m*** |cos*θ/*(| ***μ*** |^2^+| ***m*** |^2^), which can be approximated as *g* ≈ 4cos*θ*| ***m*** |/| ***μ*** | assuming | ***μ*** |>>| ***m*** |. In *C*_2_ symmetric structures such as **21**, the angle becomes 0° or 180°, which gives the maximum magnitude for the angle term. An enantiomer of **21(Ph)** slowly racemized upon heating at 90 °C in toluene at a barrier of 121 kJ mol^−1^. The helical structure of **21** was significantly extended along the helical axis by the bulky phenyl groups to increase the pitch.^[^ ^97^ ^]^ However, the structural changes did not greatly affect the optical and chiroptical properties.

**Figure 10:**
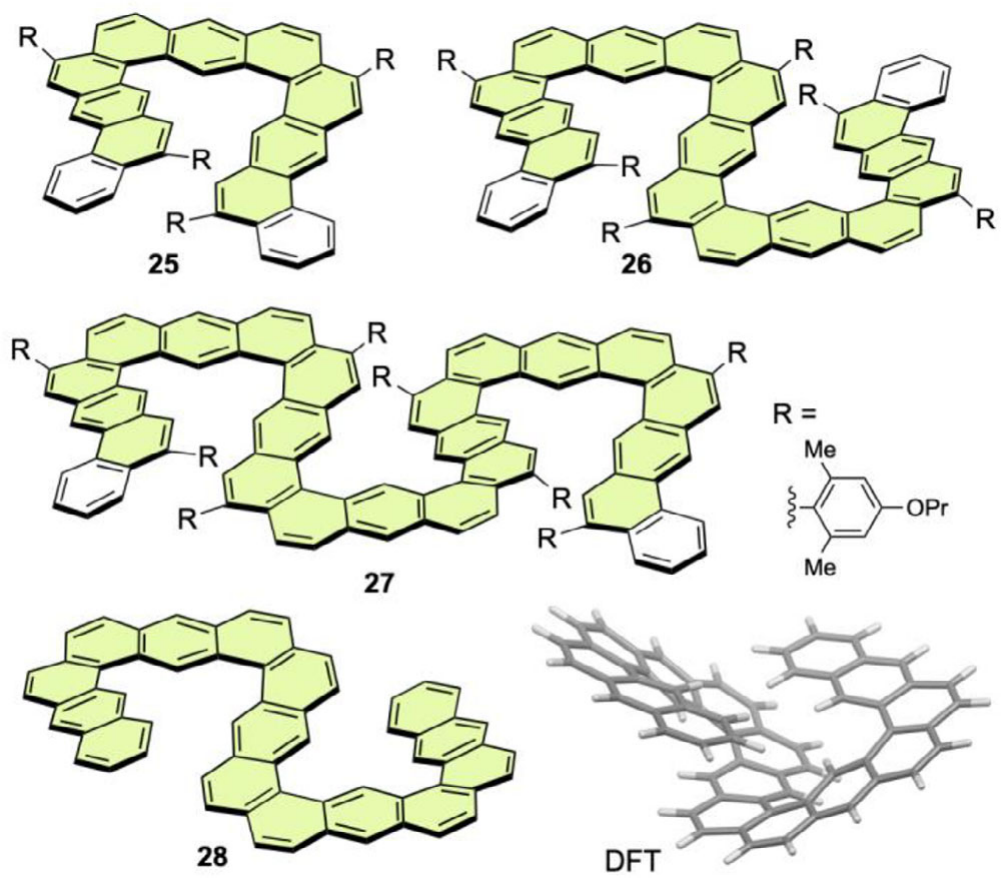
Consecutively fused single‐, double‐, and triple‐expanded helicenes.

Ikai and Yashima et al. designed single‐, double‐, and triple‐expanded helicenes **25**–**27** by sequential fusion of **[3]HA** structures (Figure 10).^[^ ^98^ ^]^ These compounds were synthesized by acid‐catalyzed alkyne annulation of anthracene‐based cyclization precursors. Compounds **26** and **27** having multiple helical moieties prefer to adopt all‐*M* or all‐*P* forms rather than other possible diastereomers. The enantiomers of these compounds were successfully resolved by chiral HPLC. While **25** (barrier 75 kJ mol^−1^) and **26** (80 kJ mol^−1^) easily racemized at 0 °C, **27** was enantiomerically stable even at 80 °C. Enantiopure samples of triple‐expanded helicene **27** showed high chiroptical performance as quantified by CPL brightness *B*_CPL_ (255 L mol^−1^ cm^−1^), an indicator of overall CPL efficiency calculated from the fluorescence quantum yield, the molar extinction coefficient, and the |*g*_lum_|.^[^ ^99^ ^]^ Recently, Toyota et al. synthesized substituent‐free compound **28** as an double‐expanded helicene.^[^ ^100^ ^]^ This compound takes a *C_i_* symmetric *M*/*P* form in a cocrystal with C_6_F_6_. In contrast, DFT calculations suggest a preference for a chiral *C*_2_ symmetric *P*/*P* (or *M*/*M*) form, in which the terminal anthracene moieties are located on the same side of the central anthracene moiety to maximize intramolecular interactions.

**Figure 11:**
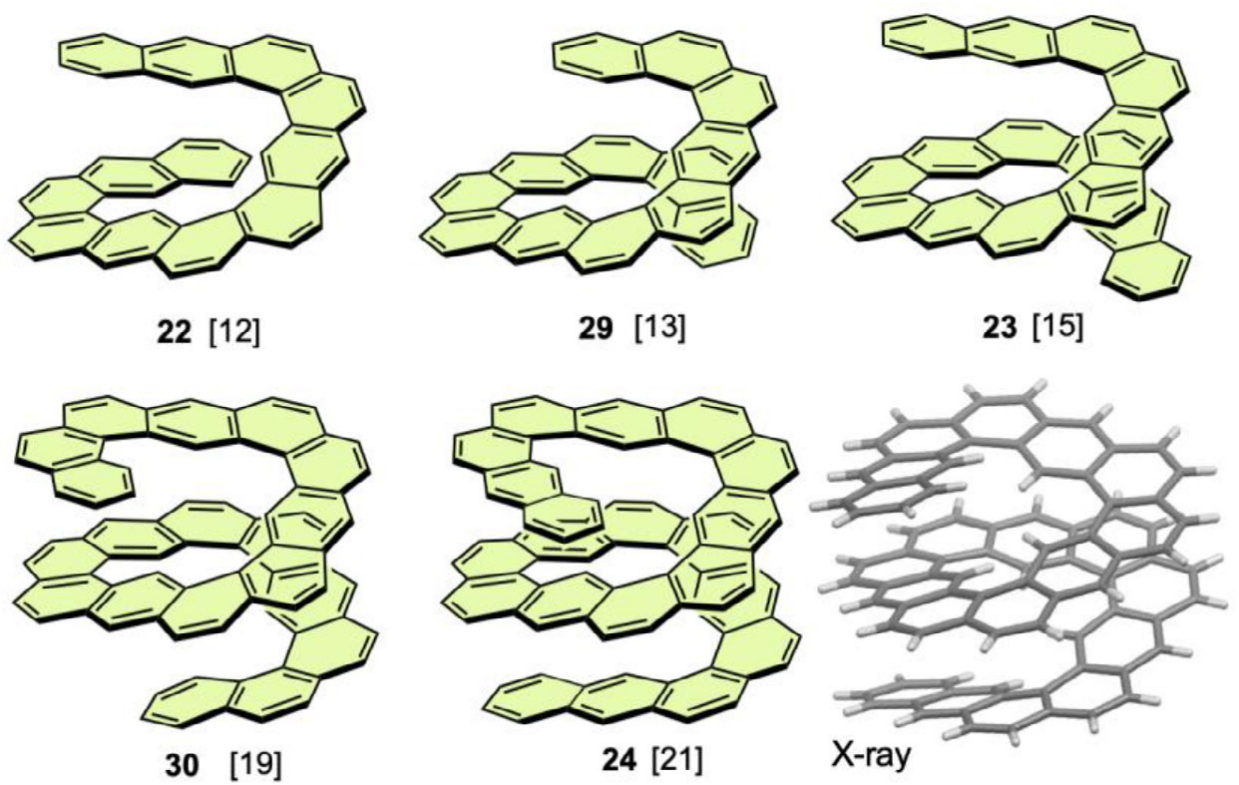
Long anthracene fused expanded helicenes. X‐ray structure of **24** is shown as stick model.

The above examples demonstrate that enantiomers of **21** can be resolved without racemization by introducing substituents or accumulating helical moieties. Another promising approach is elongation of helical chains to increase the *TN*, which should destabilize the transition state of the helical inversion process. Along this strategy, long expanded helicenes such as **22**, **23**, and **29** were synthesized (Figure 11).^[^ ^101^, ^102^ ^]^ Because the *TN* values of these compounds exceed one, a part of the aromatic moieties overlap in the helical structure with interlayer distances of 3.6–3.7 Å. In the fluorescence spectra, these compounds showed characteristic broad and long‐lived emission bands (e.g., *λ*_em_ 526 nm, *τ*_f_ 16.9 ns for **23**), suggesting excimer‐like stabilization of the excited state.^[^ ^103^ ^]^ The DFT calculations estimated the barriers to helical inversion of **29** and **23** to be 95 and 110 kJ mol^−1^, respectively, via stepwise mechanisms. The enantiomeric resolution of these compounds was unsuccessful.

To enhance the inversion barriers, longer analogs, **30** and **24**, were synthesized via four‐fold alkyne cycloisomerization.^[^ ^104^ ^]^ X‐ray analysis revealed that the helical structures of **30** (*TN* 2.03) and **24** (2.18) were highly coiled, with a part of the aromatic moieties forming triple‐layer stacking. Although the enantiomers of these compounds were resolved by chiral HPLC, facile racemization occurred for **30** at room temperature (barrier 90.7 kJ mol^−1^). The enantiomers of **24** slowly racemized at 50 °C, and its barrier (98.7 kJ mol^−1^) is higher than that of **30**. The CD and CPL spectra of enantiopure **24** were measured at room temperature without racemization. The key chiroptical data are as follows: |Δ*ε*| = 540 L mol^−1^ cm^−1^ and |*g*_abs_| = 0.032 at 400 nm for the CD spectrum and |*g*_lum_| = 0.012 at 576 nm for the CPL spectrum. It is proposed that the *C*_2_ symmetric structure, the wide inner cavity area, and the large *TN* tend to increase the *g* values of helical molecules.^[^ ^105^ ^]^ The observed data of **24** are consistent with this proposal. The mechanism of enantiomerization of **24** was analyzed using the semiempirical tight binding method (GFN2‐xTB),^[^ ^106^ ^]^ the NEB method,^[^ ^90^ ^]^ and the r^2^SCAN‐3c method.^[^ ^107^ ^]^ The calculations suggested a multistep mechanism involving inversion of each [4]helicene moiety. The calculated barrier was 110 kJ mol^−1^, which agreed well with the experimental data. Notably, the barriers to enantiomerization of such expanded helicenes were unexpectedly low, even though the *TN*s exceeded two.

**Figure 12:**
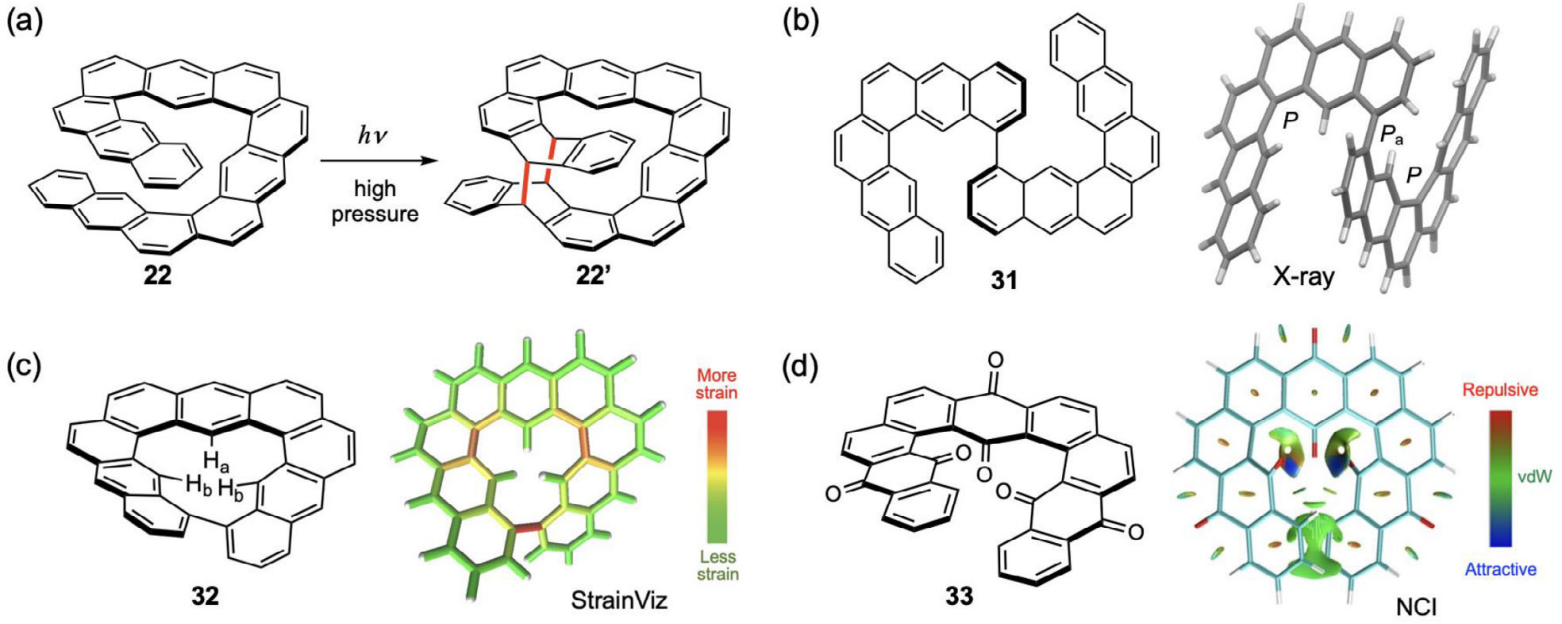
a) Pressure induced photochemical cycloaddition of **22**, b) X‐ray structure of **31**, c) StrainViz analysis of **32**, and d) NCI plot of **33**. In the StrainViz analysis, distribution of strain is displayed by color of each C–C bond. In the NCI plot, interactions are visualized by color of isosurfaces.

The effect of pressure on the photophysical properties of the helical fused anthracene derivatives was studied to explore a stimulus‐responsive system. Fluorescence spectra of **21**–**23** and **29** were measured under hydrostatic pressure up to 320 MPa.^[^ ^102^ ^]^ Ratiometric changes in fluorescence intensity were only observed for **22** at high pressure. This phenomenon is attributed to intramolecular photochemical [4 + 4]cycloaddition between stacking anthracene moieties to form **22′**, and this process is promoted under high pressure conditions (Figure 12a). Biaryl **31** (dimer of **20**) was synthesized as a new pseudohelicene with three stereogenic units, two helical moieties and one axis (Figure 12b).^[^ ^108^ ^]^ This compound exists as a mixture of three diastereomeric conformers, one of which is the *P*,*P*_a_,*P* form. The enantiomers of **31** resolved by chiral HPLC showed good performance in the CD and CPL spectra. The fluorescence spectra of racemic **31** changed significantly under hydrostatic pressure.^[^ ^109^ ^]^ The mode of spectral changes was also influenced by solvent polarity accompanied by the population changes of possible conformers. This phenomenon can be applied to a chemosensor that responds to multiple stimuli.

Another structural feature of the **[n]HA** system is significant steric congestion in the inner region. The ^1^H NMR signals of the inner H atoms shifted downfield extraordinarily: the signals due to the most deshielded protons were observed at 12.1 ppm (**21**), 12.6 ppm (**22**), 13.3 ppm (**23**), and 12.9 ppm (**24**).^[^ ^96^, ^101^, ^104^ ^]^ The steric compression effect plays an important role in the deshielding of these H atoms, as observed in sterically crowded compounds.^[^ ^110^ ^]^ In fact, the shortest nonbonded H···H distance in the inner region in **23** (ca. 1.60 Å) is much shorter than the sum of the vdW radii of H atoms (1.20 + 1.20 = 2.40 Å). The crowded environment should become severe by the locking of the helical structure by cyclization. As such a compound, **32** was synthesized by intramolecular cyclization of a derivative of **21** (Figure 12c).^[^ ^111^ ^]^ This molecule is highly strained, as indicated by the large strain energy (156 kJ mol^−1^) estimated by the StrainViz analysis.^[^ ^112^ ^]^ The shortest nonbonded H_a_···H_b_ distance was 1.65 Å, and the ^1^H NMR signal of the central inner H_a_ atom was observed at 12.5 ppm. A remarkably large nuclear Overhauser effect (NOE) of up to 44 % (theoretical limit for a homonuclear pair: 50%) was observed between the inner H signals. In the IR spectrum of **32**, a weak absorption due to the stretching vibration of the central inner C–H_a_ bond (*ν*_CH_) was observed at 3267 cm^−1^. This frequency is notably shifted to a higher frequency than those of typical aromatic C−H bonds (3000–3100 cm^−1^). These nonbonded H···H distances are classified as ultrashort contacts, and the molecular design of such compounds is a subject of interest in structural organic chemistry.^[^ ^113^, ^114^ ^]^ The CrO_3_ oxidation of **21** gave the corresponding anthraquinone derivative **33**, in which the inner C═O groups were sterically congested (Figure 12d).^[^ ^115^ ^]^ The observed C═O⋅⋅⋅C═O distance of 2.47 Å is much shorter than the sum of vdW radii of the O (1.52 Å) and C (1.70 Å) atoms. These interactions are visualized by the NCI plot. The unusual environment of the carbonyl groups was confirmed by the ^13^C NMR (upfield shift) and IR (low frequency shift) spectra.

### 3.3 Rhombic and Other Expanded Helicenes

**Figure 13:**
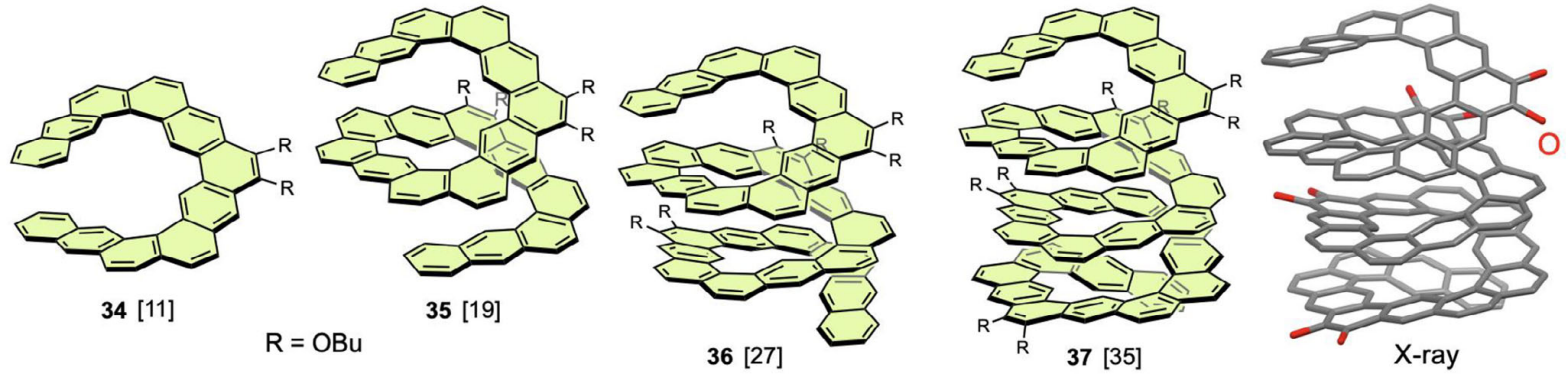
Long rhombic expanded helicenes **34**–**37** and X‐ray structure of **37** (butyl groups are omitted).

An alternative approach to the design of expanded helicene structures is the incorporation of linearly fused benzene rings to form a rhombic aromatic framework. Such expanded helicenes were reported by Isobe and Wu et al. in 2023 (Figure 13).^[^ ^116^ ^]^ Aromatic precursors were elongated via the Suzuki‐Miyaura coupling of divinylphenanthrene and diborylanthracene derivatives, and the Bi(OTf)_3_‐mediated cyclization afforded expanded helicenes **34**–**37** having 11, 19, 27, and 35 benzene rings, respectively. X‐ray analysis revealed that all of the compounds have helical structures, and the longest analog has a highly coiled structure with a *TN* of ca. 3.5. The enantiomers of **35**–**37** were resolved by chiral HPLC, and each enantiomer showed intense Cotton effects (e.g., |*g*_abs_| = 0.024 at 451 nm for **37**).

**Figure 14:**
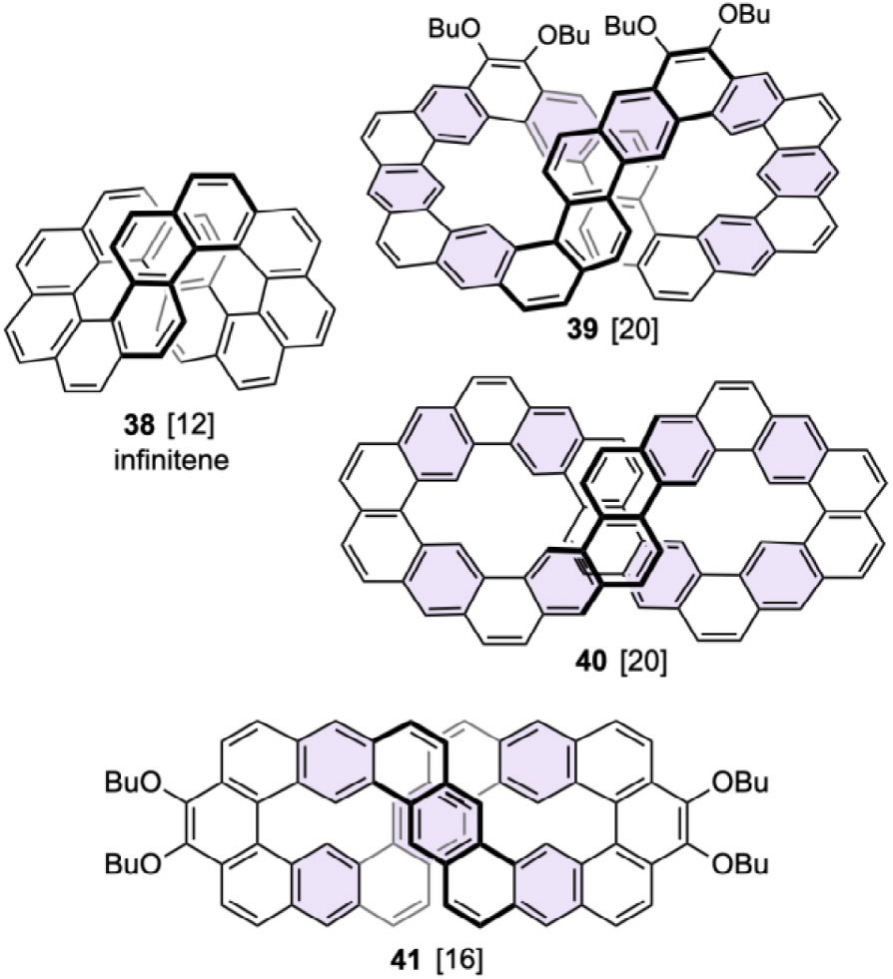
Infinitene **38** and figure‐eight compounds **39**–**41**. Linearly fused benzene rings are highlighted in light purple.

Infinitene **38**, which is a figure‐eight topological isomer of [12]circulene,^[^ ^117^, ^118^ ^]^ was synthesized by Ito and Itami et al. (Figure 14).^[^ ^119^ ^]^ Compound **39** is an extended version of infinitene having two helical moieties and eight linealy fused benzene rings. This compound with four butoxy groups was synthesized by the Bi(OTf)_3_‐mediated cyclization by Isobe and Wu et al.^[^ ^120^ ^]^ The aromatic framework of **39** was highly twisted, and the strain energy was estimated to be 143 kJ mol^−1^ by the homodesmotic reaction approach. The enantiomers of **39** resolved by chiral HPLC showed modest |*g*_abs_| values (0.005–0.007) in the CD spectra. The relative energies and various properties of the infinitenes and their expanded analogs were systematically studied by DFT calculations.^[^ ^121^ ^]^ The thermochemical stability of each compound is discussed in terms of π delocalization, π···π stacking, and steric strain. The calculations suggest that the expanded infinitene structure in **39** was less stable by 117 kJ mol^−1^ than the lowest energy isomer **40**. Another type of figure‐eight compound **41** was synthesized by Sun and Wu et al.^[^ ^122^ ^]^ This compound has a fused [5]helicene dimer with six linealy fused benzene rings. Its enantiomers showed persistent chirality and large g factors (|*g*_abs_| = 0.0054, |*g*_lum_| = 0.010).

**Figure 15:**
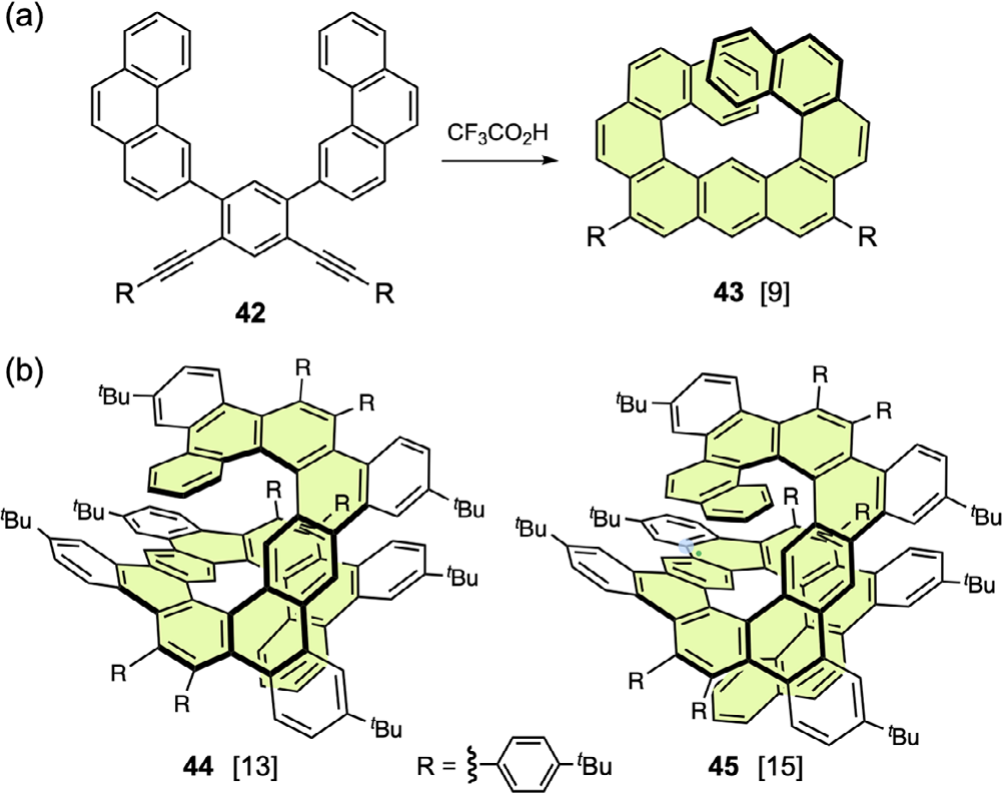
a) Formation of expanded helicene **43** by rearranged alkyne cycloisomerization of **42** and b) structures of expanded helicenes **44** and **45** prepared by enantioselective cycloaddition.

Compound **43**, a [9]helicene with one linearly fused benzene ring, was obtained via an unexpected rearrangement during acid‐catalyzed intramolecular alkyne cyclization of **42** (Figure 15a).^[^ ^123^ ^]^ This compound takes a helical structure, in which the two [5]helicene subunits have the same helicity (*P*,*P* or *M*,*M*). The performance of **43** as an organic field‐effect transistor (OFET) device was evaluated by experimental and theoretical methods. Compounds **44** and **45** with two linearly fused benzene rings were synthesized as precursors for the synthesis of highly π‐extended helicenes (Figure 15b).^[^ ^124^ ^]^ These compounds were formed by enantioselective cycloaddition of alkyne precursors. Further multiple cyclization of **44** and **45** by the Scholl reaction afforded enantiopure π‐extended [11] and [13]helicenes, respectively, which showed high CPL activity as determined by the *B*_CPL_ scale.

## 4 Other Expanded Helicenes

### 4.1 Nonhexagonal Ring Embedded Expanded Helicenes

The introduction of not only linearly fused hexagonal benzene rings but also nonhexagonal rings into the helical framework allows us to tune the helical structures and their electronic and spectroscopic properties. Broadly, these helical compounds can be regarded as expanded helicenes. This section presents some typical examples.

**Figure 16:**
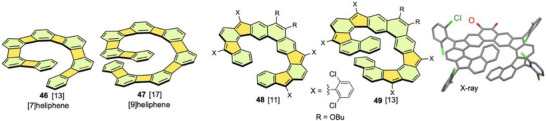
Structures of four‐membered ring‐embedded [n]heliphenes **46** and **47**, quinodimethane‐embedded expanded helicenes **48** and **49**, and X‐ray structure of **49** (butyl groups are omitted). In this section, nonhexagonal rings are highlighted in light orange, and the number of rings involving nonhexagonal rings along the helical chain is given in brackets.

Assuming that the cyclobutane moiety in biphenylene is a linearly fused ring, [n]heliphenes consisting of n benzene rings and n–1 cyclobutane rings can be regarded as expanded helicenes. Vollhardt et al. synthesized a series of [n]heliphenes using Co‐catalyzed alkyne cyclotrimerization (Figure 16).^[^ ^125^, ^126^ ^]^ As the size of the linear moieties increases, the mean helical diameter increases in the order of the original helicene (5.1 Å), heliphene (8.2 Å), and hexagonal expanded helicene (10.2 Å). Accordingly, the barrier to helical inversion decreases in the following order: [7]helicene (170 kJ mol^−1^), [7]heliphene **46** (73 kJ mol^−1^), and [2.1][13]helicene **15** (54.3 kJ mol^−1^), as analyzed by Hirose and Matsuda et al.^[^ ^43^ ^]^ The longest analog is [9]heliphene **47**, which still has a low inversion barrier (<50 kJ mol^−1^) for enantiomer resolution. It is noteworthy that the aromaticity of the nonterminal benzene rings in the [n]heliphene structures was weakened to minimize antiaromatic destabilization.

Chi et al. designed quinodimethane‐embedded extended [11]helicene **48** and [13]helicene **49** with four five‐membered rings and two linearly fused benzene rings (Figure 16).^[^ ^127^ ^]^ These compounds were obtained by oxidative aromatization of the corresponding tetrahydro precursors with chloranil. The helical structures of these expanded helicenes were confirmed by X‐ray analysis, and the terminal benzene rings in **49** overlap significantly at a separation of 3.4 Å. The ^1^H NMR measurements showed that the helical inversion in **49** occurred slowly on the NMR timescale (barrier 76 kJ mol^−1^), whereas this process occurred rapidly for **48** (calcd. barrier 38 kJ mol^−1^). These compounds can be chemically oxidized to radical cations and dications, or chemically reduced to radical anions and dianions. The dications and dianions exhibit significant open‐shell singlet diradical character, because their singlet–triplet energy gaps are smaller than that of their original neutral compounds.

**Figure 17:**
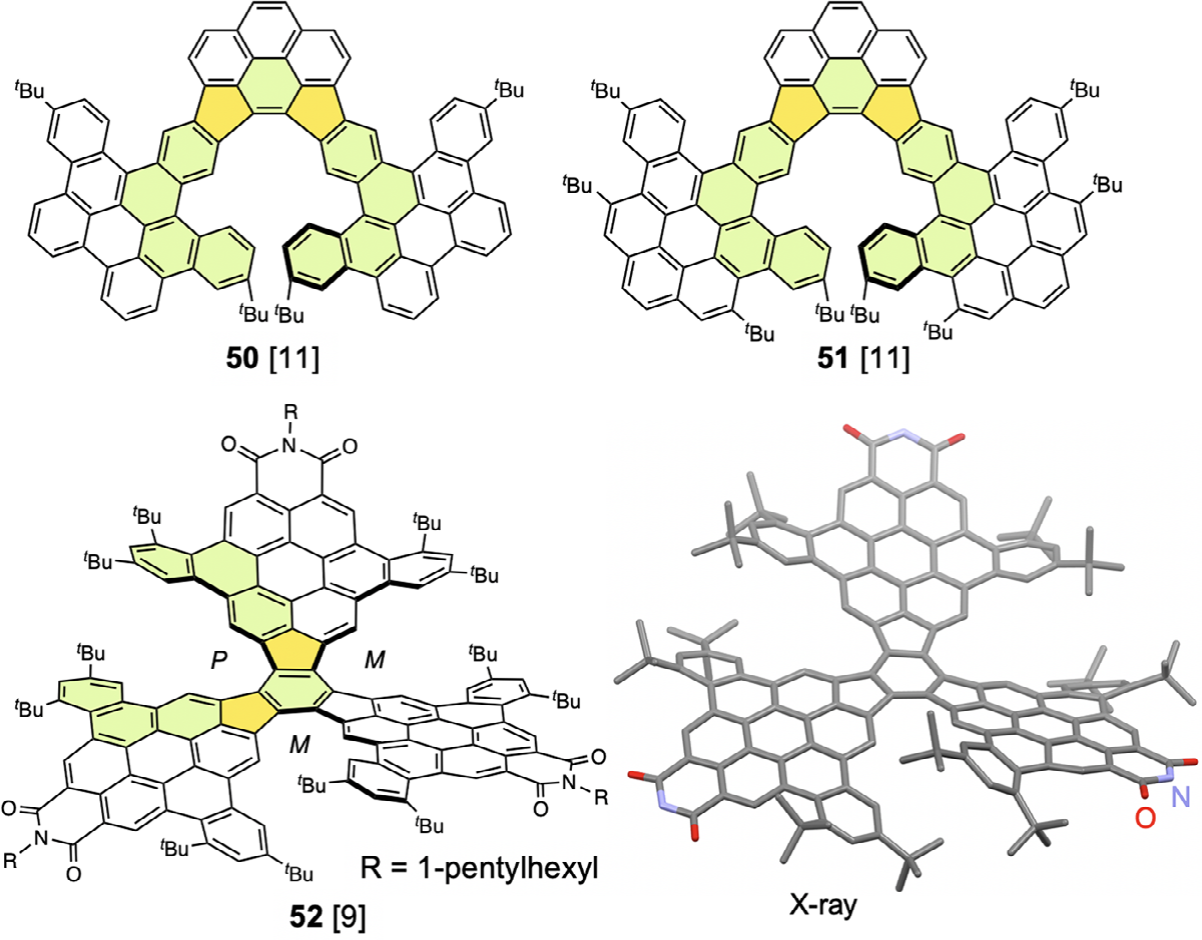
Structures of pentagon‐embedded expanded helicenes **50**–**52** and X‐ray structure of **52** (*N*‐(1‐pentylhexyl) groups are omitted). In the structure of triple‐expanded helicene **52**, rings in only one helical chain are highlighted.

Xiao et al. designed pentagon‐embedded expanded and π‐extended [11]helicenes **50** and **51** with two five‐membered rings and two linearly fused benzene rings (Figure 17).^[^ ^128^ ^]^ These compounds were synthesized by double cyclization to form two five‐membered rings. DFT calculations revealed that these compounds had helical structures along eleven rings, and their estimated barriers to enantiomerization were >170 kJ mol^−1^. Their fluorescence spectra showed intense red emission at ca. 620 nm. These expanded helicenes can be chemically oxidized by AgSbF_6_ to form stable radical cations, as confirmed by near‐IR (NIR) and electron paramagnetic resonance (EPR) spectra. These compounds can be used as radiative cooling materials in a glass model house. Compound **52** was synthesized as an expanded helicene containing three dibenzocoronene monoimide structures.^[^ ^129^ ^]^ Each helical moiety contains two pentagons and two linearly fused benzene rings with a central benzene ring shared by three helical moieties. X‐ray analysis revealed that the molecule took a *C*_1_ symmetric nonpropeller form instead of a *C*_3_ symmetric propeller form. The ^1^H NMR spectrum of **52** gave several signals at room temperature, which were simplified at 120 °C. This dynamic process is attributed to the exchange between possible conformers via twist‐to‐propeller conversions, which occur on the NMR timescale at high temperatures (barrier 64 kJ mol^−1^).

### 4.2 Expanded Heterohelicenes

The incorporation of heteroatoms into expanded helicene frameworks significantly impacts the structure and properties owing to the electronic demands of noncarbon atoms. For instance, several helicenes and expanded helicenes containing N atoms have been developed as CPL‐active compounds.^[^ ^130^ ^]^ Recent typical examples containing N, B, O, and S atoms are presented in this section.

**Figure 18:**
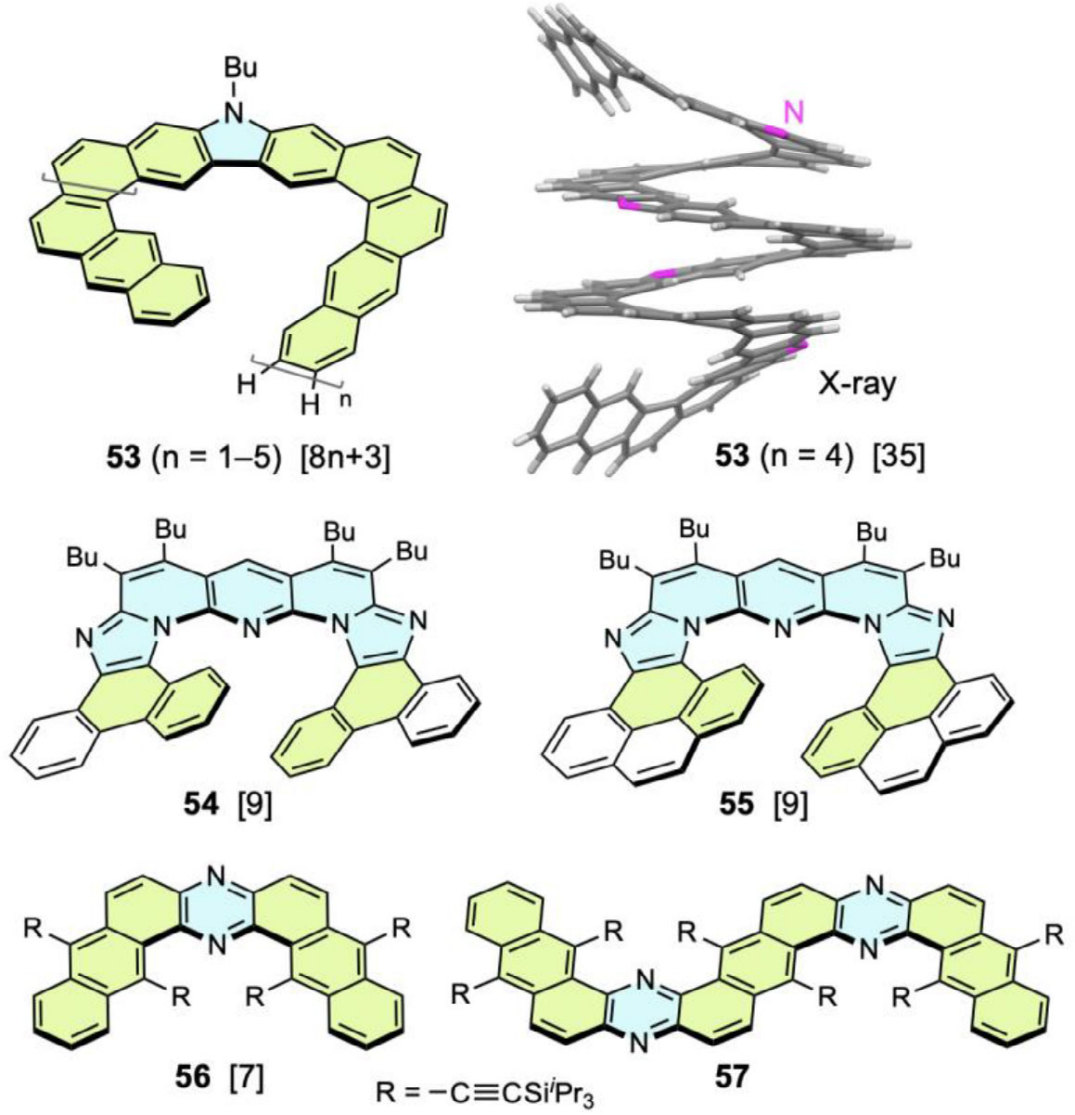
N‐embedded expanded helicenes **53**–**57**. In the X‐ray structure of **53** (n = 4), *N*‐butyl groups are omitted. Heteroaromatic rings are highlighted in light blue in this section.

Yang and Wu et al. synthesized expanded azahelicenes **53** (n = 1–5) from carbazole and anthracene units by a method similar to that used for the synthesis of expanded carbohelicenes **34**–**37** (Figure 18).^[^ ^131^ ^]^ The helical structures were confirmed by X‐ray analysis or DFT calculations. The longest analog **53** (n = 5) consists of 43 fused rings with a *TN* close to 4. The enantiomers of **53** (n = 2, 3, and 4) were resolved by chiral HPLC and exhibited good chiroptical properties (|*g*_abs_| = 0.043 and |*g*_lum_| = 0.021 for n = 4). Choudhury et al. proposed the molecular design of conformationally flexible heterohelicenes **54** and **55**, which behave like molecular springs.^[^ ^132^ ^]^ The helicene framework was expanded by adding one linearly fused pyridine unit to two angularly fused pyridine units and two five‐membered imidazole units. These compounds had helical structures with stacking of the terminal benzene rings, in which the centroid‐to‐centroid distances were 3.66 Å for **54** and 3.77 Å for **55**. The inversion barrier of **55** was estimated to be ca. 76 kJ mol^−1^. The UV‐vis and fluorescence spectra of the imidazole derivatives were reversibly affected by protonation and deprotonation, resulting in a pH‐responsive system. This phenomenon is regarded as a reversible switching of stimuli‐responsive soft molecular springs. Mateo‐Alonso et al. synthesized pyrazine‐embedded expanded helicenoids **56** and **57**.^[^ ^133^, ^134^ ^]^ Because of the presence of bulky substituents, each half‐helix moiety (*TN* = ca. 0.5) takes a twisted structure. For example, the torsion angles along the two [3]helicene substructures are ca. 20° in **56**. Compound **57** takes a highly distorted aromatic framework in an achiral *meso* form in the crystal, where the two helical moieties have *P* and *M* helicity.

**Figure 19:**
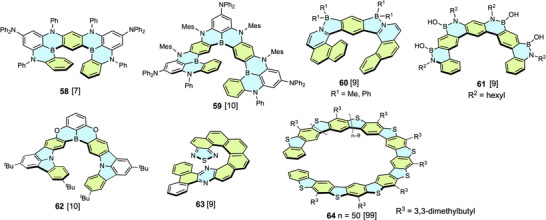
B, N, O, or S‐embedded expanded helicene **58**–**63**.

Recently, BN‐embedded helicenes have attracted attention due to advances in synthetic methods and their unique photophysical properties.^[^ ^135^, ^136^, ^137^ ^]^ Hatakeyama et al. synthesized BN‐embedded compound **58** by borylation (Figure 19).^[^ ^138^ ^]^ This compound is a thermally activated delayed‐fluorescence (TADF) material that shows a very sharp blue emission (full width at half maximum [FWHM] 14 nm). This blue emitter exhibited excellent organic light‐emitting diode (OLED) device performance with an external quantum efficiency (EQE) of up to 34.4%. The framework of **58** was utilized for the development of OLED devices by modifying the substituents and heteroatoms.^[^ ^139^, ^140^ ^]^ Hatakeyama et al. also synthesized compound **59** consisting of three BN_2_‐embedded [4]helicene units and two terminal benzene rings by one‐shot triple borylation of an oligoaniline precursor with BBr_3_ in an autoclave.^[^ ^141^ ^]^

Nowak‐Król et al. synthesized [9]hetelohelicenes **60** with two azaborole rings and one linearly fused benzene ring.^[^ ^142^ ^]^ In the helical structures of **60**, the terminal benzene rings are distorted by 28–50° relative to the central benzene ring, forming a highly deformed structure. The DFT calculation suggested that the helical inversion of **60** (R^1^ = Me) requires a low barrier of ca. 60 kJ mol^−1^ via a multistep mechanism. Compound **60** (R^1^ = Ph) exhibited intense blue fluorescence (*Φ*_f_ 0.47) in CH_2_Cl_2_ solution and green fluorescence (*Φ*_f_ 0.25) in the solid state. Another example of BN‐embedded expanded helicenes is compound **61**, which was developed for a material for OFET memory.^[^ ^143^ ^]^ This compound functions as a molecular floating gate for ambipolar charge trapping memory. Zhang and Duan et al. synthesized BNO‐containing expanded helicene **62** with two linearly fused benzene rings.^[^ ^144^ ^]^ This compound was also highly emissive with a small band width, and a device based on this compound demonstrated superior organic electroluminescence (OEL) performance.

Two examples of S‐containing expanded helicenes are shown in Figure 19. In order to adjust the electronic distribution, compound **63** containing a 2,1,3‐thiadiazole ring and a phenanthrene‐fused pyrazine ring was designed.^[^ ^145^ ^]^ Due to the permanent polarity and wide π‐surface, enantiopure molecules form face‐to‐face homochiral columnar assemblies via strong intermolecular interactions. This compound exhibited large photoconductivity in the solid state, indicating its potential application as an organic semiconductor. Compound **64** consists of 44 thiophene rings and 45 benzene rings,^[^ ^146^ ^]^ namely an expanded analog of thiahelicene.^[^ ^147^, ^148^ ^]^ The *TN* of this very long expanded heterohelicene is ca. 5.8 and its length is ca. 2 nm. Enantiopure samples of **63** exhibited strong mirror‐image CD, and this activity was retained upon heating at 200 °C for 10 h.

## 5 Summary and Outlook

Expanded helicenes were originally defined as helicenes composed of alternating linearly and angularly fused rings. However, this definition has been extended to include helicenes with any number, type, or combination of linearly fused rings. Following Tilley's first report of hexagonal expanded helicenes in 2017, various expanded helicenes have been synthesized from fused aromatic units to construct exceptional helical structures. The size and shape of expanded helicenes can be modified through the arrangement of linearly fused rings, and they can be classified into hexagonal, triangular, rhombic, and other shapes, characterized by several index systems. The molecular structures of expanded helicenes are discussed in terms of helical diameters, pitches, and *TN*s, similar to those of mechanical springs. Expanded helicenes have large helical diameters, an inner cavity surrounded by H atoms, and soft spring‐like flexibility. For expanded helicenes with large *TN*, π···π interactions between helical chains at ca. 3.4 Å are important to stabilize the helical structures. Chirality is a key issue in the chemistry of expanded helicenes. Enantiomers can be resolved if the helical chain is sufficiently elongated to enhance the barrier to racemization through helical inversion in expanded helicenes with flexible structures. The mechanism of helical inversion is determined by theoretical methods, where the enantiomerization process usually proceeds through the stepwise inversion of helicene substructures. Several expanded helicenes have been resolved into their enantiomers using chiral HPLC, and some of them exhibit high chiroptical activity in the CD and CPL spectra, which is necessary for the development of chiral functional materials. Although the correlation between structure and chiroptical properties is not yet been fully understood, recent studies have shed light on the topic. Expanded helicenes are attractive models of nanoscale molecular springs,^[^ ^149^ ^]^ and their mechanical performance has been studied theoretically by calculating their pitches and force constants. The potential of expanded helicenes has been further enhanced by the incorporation of nonhexagonal rings and heteroaromatic rings. Because of their diverse molecular designs, expanded helicenes and other helicene families are promising structures for the development of functional π‐conjugated materials, such as electronic devices, optical sensors, and FL and CPL emitters. The chemistry of expanded helicenes is an exciting field that continues to expand the horizon of unknown molecules and their properties in both basic and applied sciences.

## Conflict of Interest

The author declare no conflict of interest.

## Data Availability

Data sharing is not applicable to this article as no new data were created or analyzed in this study.

## References

[1] R. H. Martin , Angew. Chem. Int. Ed. Engl. 1974, 13, 649.

[2] Y. Shen , C.‐F. Chen , Chem. Rev. 2012, 112, 1463. 22017405 10.1021/cr200087r

[3] M. Gingras , Chem. Soc. Rev. 2013, 42, 968. 23151799 10.1039/c2cs35154d

[4] J. Crassous , I. G. Stará , I. Starý , Eds., Helicenes: Synthesis, Properties and Applications, Wiley‐VCH, Weinheim, 2022.

[5] C.‐F. Chen , Y. Shen , Eds., Helicene Chemistry: From Synthesis to Applications, Springer, Berlin, 2017.

[6] I. G. Stará , I. Starý , in Molecular Nanographenes: Synthesis, Properties, and Applications, (Eds.: N. Martin , C. Nuckolls ), Wiley‐VCH, Weinheim, 2025, Chap. 5.

[7] IUPAC Compendium of Chemical Terminology, 2nd ed. (the "Gold Book"). Compiled by A. D. McNaught , A. Wilkinson , Eds. Blackwell Scientific Publications, Oxford, 1997. Online version can be found under https://goldbook.iupac.org, (accessed 3 July 2025).

[8] G. P. Moss , P. A. S. Smith , D. Tavernier , Pure Appl. Chem. 1995, 67, 1307.

[9] G. P. Moss , Pure Appl. Chem. 1996, 68, 2193.

[10] M. S. Newman , W. B. Lutz , D. Lednicer , J. Am. Chem. Soc. 1955, 77, 3420.

[11] M. S. Newman , D. Lednicer , J. Am. Chem. Soc. 1956, 78, 4765.

[12] D. A. Lightner , D. T. Hefelfinger , G. W. Frank , T. W. Powers , K. N. Trueblood , Nature (London), Phys. Sci. 1971, 232, 124.4933245

[13] D. A. Lightner , D. T. Hefelfinger , T. W. Powers , G. W. Frank , K. N. Trueblood , J. Am. Chem. Soc. 1972, 94, 3492.

[14] M. Gingras , Chem. Soc. Rev. 2013, 42, 1051. 23151680 10.1039/c2cs35134j

[15] M. Gingras , G. Félix , R. Peresutti , Chem. Soc. Rev. 2013, 42, 1007. 23151610 10.1039/c2cs35111k

[16] K.‐H. Ernst , Acc. Chem. Res. 2016, 49, 1182. 27251099 10.1021/acs.accounts.6b00110

[17] M. Yamaguchi , M. Arisawa , M. Shigeno , N. Saito , Bull. Chem. Soc. Jpn. 2016, 89, 1145.

[18] N. Saleh , C. Shen , J. Crassous , Chem. Sci. 2014, 5, 3680.

[19] G. Zhang , J. Zhang , Y. Tao , F. Gan , G. Lin , J. Liang , C. Shen , Y. Zhang , H. Qiu , Nat. Commun. 2024, 15, 5469. 38937477 10.1038/s41467-024-49865-y PMC11211482

[20] L. Rulíšek , O. Exner , L. Cwiklik , P. Jungwirth , I. Starý , L. Pospíšil , Z. Havlas , J. Phys. Chem. C 2007, 111, 14948.

[21] W.‐B. Lin , M. Li , L. Fang , C.‐F. Chen , Chin. Chem. Lett. 2018, 29, 40.

[22] V. V. Porsev , R. A. Evarestov , Nanomaterials 2023, 13, 2295. 36770376 10.3390/nano13030415 PMC9920107

[23] V. V. Porsev , A. V. Bandura , R. A. Evarestov , Comput. Mater. Sci. 2022, 203, 111063.

[24] V. V. Porsev , A. D. Bandura , S. I. Lukyanov , R. A. Evarestov , Carbon 2019, 152, 755.

[25] X. Liu , X. Cui , X. Zhang , J.‐P. Wu , C. Shen , Theor. Chem. Acc. 2024, 143, 17.

[26] P. Šesták , J. Wu , J. He , J. Pokluda , Z. Zhang , Phys. Chem. Chem. Phys. 2015, 17, 18684. 26118679 10.1039/c5cp02043c

[27] R. H. Martin , M. Baes , Tetrahedron 1975, 31, 2135.

[28] P. Sehnal , I. G. Stará , D. Šaman , I. Starý , Proc. Natl. Acad. Sci. USA 2009, 106, 13169. 19633186 10.1073/pnas.0902612106 PMC2726423

[29] K. Mori , T. Murase , M. Fujita , Angew. Chem. Int. Ed. 2015, 54, 6847. 10.1002/anie.201502436 25907312

[30] G. R. Kiel , S. C. Patel , P. W. Smith , D. S. Levine , T. D. Tilley , J. Am. Chem. Soc. 2017, 139, 18456. 29215272 10.1021/jacs.7b10902

[31] L.‐J. Peng , X.‐Y. Wang , Z.‐A. Li , H.‐Y. Gong , Asian J. Org. Chem. 2023, 12, e202300543.

[32] H. V. Anderson , N. D. Gois , W. A. Chalifoux , Org. Chem. Front. 2023, 10, 4167.

[33] Y. Zhu , J. Wang , Acc. Chem. Res. 2023, 56, 363. 36700652 10.1021/acs.accounts.2c00767

[34] Y. Zhang , J. Guan , L. Luo , X. Han , J. Wang , Y. Zheng , J. Xu , Interdiscip. Mater. 2024, 3, 453.

[35] P. Izquierdo‐García , J. M. Fernández‐García , S. M. Rivero , M. Šámal , J. Rybáček , L. Bednárová , S. Ramírez‐Barroso , F. J. Ramírez , R. Rodríguez , J. Perles , D. García‐Fresnadillo , J. Crassous , J. Casado , I. G. Stará , N. Martín , J. Am. Chem. Soc. 2023, 145, 11599. 37129470 10.1021/jacs.3c01088 PMC10236438

[36] Y. Nakakuki , T. Hirose , H. Sotome , H. Miyasaka , K. Matsuda , J. Am. Chem. Soc. 2018, 140, 4317. 29551070 10.1021/jacs.7b13412

[37] C. M. Cruz , S. Castro‐Fernández , E. Maçôas , J. M. Cuerva , A. G. Campaña , Angew. Chem. Int. Ed. 2018, 57, 14782. 10.1002/anie.201808178 30144368

[38] Y. Chen , C. Lin , Z. Luo , Z. Yin , H. Shi , Y. Zhu , J. Wang , Angew. Chem. Int. Ed. 2021, 60, 7796. 10.1002/anie.202014621 33410247

[39] Y.‐J. Shen , L.‐J. Peng , L.‐N. Diao , N.‐T. Yao , W.‐K. Chen , Y. Yang , M. Qiu , W.‐X. Zhu , X. Li , X.‐Y. Wang , H.‐Y. Gong , Org. Lett. 2024, 26, 7279. 39024649 10.1021/acs.orglett.4c02093

[40] Z.‐A. Li , K.‐L. Zhu , N.‐T. Yao , J. Liang , Y.‐L. Shang , Y. Zhang , H.‐Y. Gong , Chem. Sci. 2025, 16, 9978. 40336997 10.1039/d5sc01498k PMC12053474

[41] R. G. Harvey , Polycyclic Aromatic Hydrocarbons, Wiley‐VCH, New York, 1997, Chap. 6.

[42] S. W. Slayden , J. F. Liebman , Chem. Rev. 2001, 101, 1541. 11710232 10.1021/cr990324+

[43] Y. Nakakuki , T. Hirose , K. Matsuda , J. Am. Chem. Soc. 2018, 140, 15461. 30339380 10.1021/jacs.8b09825

[44] T. Mori , Chem. Rev. 2021, 121, 2373. 33411513 10.1021/acs.chemrev.0c01017

[45] C. Li , Y. Yang , Q. Miao , Chem. Asian J. 2018, 13, 884. 29432658 10.1002/asia.201800073

[46] Y.‐F. Wu , L. Zhang , Q. Zhang , S.‐Y. Xie , L.‐S. Zheng , Org. Chem. Front. 2022, 9, 4726.

[47] A. Tsurusaki , K. Kamimawa , Chem. Lett. 2021, 50, 1913.

[48] H.‐C. Huang , Y.‐C. Hsieh , P.‐L. Lee , C.‐C. Lin , Y.‐S. Ho , W.‐K. Shao , C.‐T. Hsieh , M.‐J. Cheng , Y.‐T. Wu , J. Am. Chem. Soc. 2023, 145, 10304. 37099267 10.1021/jacs.3c01647

[49] Z. Chen , W.‐C. Guo , C.‐F. Chen , Org. Biomol. Chem. 2025, 23, 7501, Advance Article, DOI: 10.1039/D5OB00966A. 40704784

[50] Z. Gan , J. Lai , L. Lai , S.‐Y. Xie , Q. Zhang , Adv. Optical Mater. 2025, 13, e00976, Early View, DOI: 10.1002/adom.20250097.

[51] M. Cei , L. Di Bari , F. Zinna , Chirality 2023, 35, 192. 36707940 10.1002/chir.23535

[52] H. Tanaka , Y. Inoue , T. Mori , ChemPhotoChem 2018, 2, 386.

[53] V. Kumar , J. L. Páez , S. Míguez‐Lago , J. M. Cuerva , C. M. Cruz , A. G. Campaña , Chem. Soc. Rev. 2025, 54, 4922. 40208628 10.1039/d4cs00745j

[54] C. Duan , H. Xin , X. Gao , Tetrahedron Lett. 2023, 123, 154553.

[55] I. A. S. Chaolumen , K. E. Yamada , H. Ito , K. Itami , Angew. Chem. Int. Ed. 2021, 60, 23508. 10.1002/anie.202100260 33547701

[56] X. Yang , M. Hoffmann , F. Rominger , T. Kirschbaum , A. Dreuw , M. Mastalerz , Angew. Chem. Int. Ed. 2019, 58, 10650. 10.1002/anie.201905666 31125478

[57] Y.‐Y. Ju , H. Luo , Z.‐J. Li , B.‐H. Zheng , J.‐F. Xing , X.‐W. Chen , L.‐X. Huang , G.‐H. Nie , B. Zhang , J. Liu , Y.‐Z. Tan , Angew. Chem. Int. Ed. 2024, 63, e202402621. 10.1002/anie.202402621 38443314

[58] Y. Fei , J. Liu , Adv. Sci. 2022, 9, 2201000. 10.1002/advs.202201000 PMC9259726 35470978

[59] C. M. Cruz , A. G. Campaña , Synlett 2024, 35, 1480.

[60] Z. Qiu , S. Asako , Y. Hu , C.‐W. Ju , T. Liu , L. Rondin , D. Schollmeyer , J.‐S. Lauret , K. Müllen , A. Narita , J. Am. Chem. Soc. 2020, 142, 14814. 32809808 10.1021/jacs.0c05504 PMC7472433

[61] W.‐W. Yang , J.‐J. Shen , Chem. Eur. J. 2022, 28, e202202069. 35951443 10.1002/chem.202202069

[62] K. Dhbaibi , L. Favereau , J. Crassous , Chem. Rev. 2019, 119, 8846. 31294973 10.1021/acs.chemrev.9b00033

[63] C. Maeda , K. Nagahata , T. Shirakawa , T. Ema , Angew. Chem. Int. Ed. 2020, 59, 7813. 10.1002/anie.202001186 32107825

[64] V. Kumar , S. D. Dongre , G. Venugopal , A. Narayanan , S. S. Babu , Chem. Commun. 2024, 60, 11944. 10.1039/d4cc03707c 39352689

[65] A. Nowak‐Król , P. T. Geppert , K. R. Naveen , Chem. Sci. 2024, 15, 7408. 38784742 10.1039/d4sc01083c PMC11110153

[66] Y. Matsuo , M. Gon , K. Tanaka , S. Seki , T. Tanaka , J. Am. Chem. Soc. 2024, 146, 17428. 38866732 10.1021/jacs.4c05156

[67] M. Qui , J. Du , N.‐T Yao , X.‐Y. Wang , H.‐Y. Gong , Beilstein J. Org. Chem. 2025, 21, 1422. 40661750 10.3762/bjoc.21.106 PMC12256785

[68] T. W. Bell , H. Jousselin , J. Am. Chem. Soc. 1991, 113, 6283.

[69] I. Gutman , Theor. Chim. Acta 1977, 45, 309.

[70] F. Khaleel , S. Chakraborty , R. Gershoni‐Poranne , J. Phys. Org. Chem. 2025, 38, e70012.

[71] R. Gershoni‐Poranne , Chem. Eur. J. 2018, 24, 4165. 29315938 10.1002/chem.201705407

[72] A. T. Balaban , Tetrahedron 1969, 25, 2949.

[73] A. T. Balaban , M. Pompe , J. Phys. Chem. A 2007, 111, 2448. 17388312 10.1021/jp068743f

[74] I. Agranat , B. A. Hess , L. J. Schaad , Pure Appl. Chem. 1980, 52, 1399.

[75] F. Xu , H. Yu , A. Sadrzadeh , B. I. Yakobson , Nano Lett. 2016, 16, 34. 26452145 10.1021/acs.nanolett.5b02430

[76] S. J. Cyvin , J. Brunvoll , B. N. Cyvin , Theory of Coronoid Hydrocarbons, Springer, Berlin, 1991.

[77] J. Brunvoll , B. N. Cyvin , S. J. Cyvin , J. Chem. Inf. Sci. 1987, 27, 14.

[78] R. H. Martin , M. J. Marchant , Tetrahedron 1974, 30, 347.

[79] R. H. Janke , G. Haufe , E.‐U. Würthwein , J. H. Borkent , J. Am. Chem. Soc. 1996, 118, 6031.

[80] J. Barroso , J. L. Cabellos , S. Pan , F. Murillo , X. Zarate , M. A. Fernandez‐Herrera , G. Merino , Chem. Commun. 2018, 54, 188. 10.1039/c7cc08191j 29220058

[81] L.‐j. Gong , C.‐y. Liu , C. Ma , W.‐f. Lin , J.‐k. Lv , X.‐y. Zhang , RSC Adv. 2019, 9, 17382. 35519869 10.1039/c9ra01136f PMC9064566

[82] A. E. Samkian , G. R. Kiel , C. G. Jones , H. M. Bergman , J. Oktawiec , H. M. Nelson , T. D. Tilley , Angew. Chem. Int. Ed. 2021, 60, 2493. 10.1002/anie.202012213 33090649

[83] G. R. Kiel , H. M. Bergman , A. E. Samkian , N. J. Schuster , R. C. Handford , A. J. Rothenberger , R. Gomez‐Bombarelli , C. Nuckolls , T. D. Tilley , J. Am. Chem. Soc. 2022, 144, 23421. 36525313 10.1021/jacs.2c09555

[84] G. R. Kiel , K. L. Bay , A. E. Samkian , N. J. Schuster , J. B. Lin , R. C. Handford , C. Nuckolls , K. N. Houk , T. D. Tilley , J. Am. Chem. Soc. 2020, 142, 11084. 32450694 10.1021/jacs.0c03177

[85] P. Ravat , Chem. Eur. J. 2021, 27, 3957. 33034405 10.1002/chem.202004488 PMC7986117

[86] M. Reist , B. Testa , P.‐A. Carrupt , M. Jung , V. Schurig , Chirality 1995, 7, 396.

[87] J. Poater , M. Duran , M. Solà , Front. Chem. 2018, 6, 561. 30515378 10.3389/fchem.2018.00561 PMC6255896

[88] T. M. Krygowski , M. K. Cyrański , Chem. Rev. 2001, 101, 1385. 11710226 10.1021/cr990326u

[89] S. Suárez‐Pantiga , P. Redero , X. Aniban , M. Simon , C. Golz , R. A. Mata , M. Alcarazo , Chem. Eur. J. 2021, 27, 13358. 34288171 10.1002/chem.202102585 PMC8519012

[90] G. Henkelman , B. P. Uberuaga , H. Jónsson , J. Chem. Phys. 2000, 113, 9901.

[91] M. Toya , T. Omine , F. Ishiwari , A. Saeki , H. Ito , K. Itami , J. Am. Chem. Soc. 2023, 145, 11553. 37202849 10.1021/jacs.3c00109

[92] R. A. Boto , F. Peccati , R. Laplaza , C. Quan , A. Carbone , J.‐P. Piquemal , Y. Maday , J. Contreras‐García , J. Chem. Theory Comput. 2020, 16, 4150. 32470306 10.1021/acs.jctc.0c00063

[93] J. M. Fernández‐García , P. Izquierdo‐García , M. Buendía , S. Filippone , N. Martín , Chem. Commun. 2022, 58, 2634. 10.1039/d1cc06561k 35139140

[94] M. Daigle , D. Miao , A. Lucotti , M. Tommasini , J.‐F. Morin , Angew. Chem. Int. Ed. 2017, 56, 6213. 10.1002/anie.201611834 28267293

[95] T. Lukmanov , A. F. Akhmetov , D. S. Sabirov , *C* 2022, 8, 61.

[96] K. Fujise , E. Tsurumaki , G. Fukuhara , N. Hara , Y. Imai , S. Toyota , Chem. Asian J. 2020, 15, 2456. 32573111 10.1002/asia.202000394

[97] K. Suzuki , H. Fukuda , H. Toda , Y. Imai , Y. Nojima , M. Hasegawa , E. Tsurumaki , S. Toyota , Tetrahedron 2023, 132, 133243.

[98] W. Zheng , T. Ikai , K. Oki , E. Yashima , Nat. Sci. 2022, 2, e20210047.

[99] L. Arrico , L. Di Bari , F. Zinna , Chem. Eur. J. 2021, 27, 2920. 32725832 10.1002/chem.202002791

[100] T. Kihara , Y. Watanabe , E. Tsurumaki , M. Yamashina , S. Toyota , Can. J. Chem. **2025**, e‐First. 10.1139/cjc-2025-0093.

[101] K. Fujise , E. Tsurumaki , K. Wakamatsu , S. Toyota , Chem. Eur. J. 2021, 27, 4548. 33205503 10.1002/chem.202004720

[102] T. Kinoshita , K. Fujise , E. Tsurumaki , S. Toyota , G. Fukuhara , Chem. Commun. 2022, 58, 3290. 10.1039/d2cc00428c 35175268

[103] M. Buchta , J. Rybáček , A. Jančařík , A. A. Kudale , M. Buděšínský , J. V. Chocholoušová , J. Vacek , L. Bednárová , I. Císařová , G. J. Bodwell , I. Starý , I. G. Stará , Chem. Eur. J. 2015, 21, 8910. 25925496 10.1002/chem.201500826

[104] H. Fukuda , M. Kobayashi , E. Tsurumaki , M. Yamashina , M. Hasegawa , K. Wakamatsu , S. Toyota , Chem. Eur. J. 2025, 31, e202404348. 39664000 10.1002/chem.202404348

[105] R. G. Uceda , C. M. Cruz , S. Míguez‐Lago , L. Álvarez de Cienfuegos , G. Longhi , D. A. Pelta , P. Novoa , A. J. Mota , J. M. Cuerva , D. Miguel , Angew. Chem. Int. Ed. 2024, 63, e202316696. 10.1002/anie.202316696 38051776

[106] C. Bannwarth , S. Ehlert , S. Grimme , J. Chem. Theory Comput. 2019, 15, 1652. 30741547 10.1021/acs.jctc.8b01176

[107] S. Grimme , A. Hansen , S. Ehlert , J.‐M. Mewes , J. Chem. Phys. 2021, 154, 064103. 33588555 10.1063/5.0040021

[108] K. Watanabe , E. Tsurumaki , M. Hasegawa , S. Toyota , Chem. Eur. J. 2024, 30, e202400929. 38554080 10.1002/chem.202400929

[109] T. Kinoshita , K. Watanabe , E. Tsurumaki , S. Toyota , G. Fukuhara , Chem. Commun. 2025, 61, 1124. 10.1039/d4cc05652c 39648994

[110] B. V. Cheney , J. Am. Chem. Soc. 1968, 90, 5386.

[111] H. Fukuda , E. Tsurumaki , K. Wakamatsu , S. Toyota , Chem. Eur. J. 2024, 30, e202401627. 38751350 10.1002/chem.202401627

[112] C. E. Colwell , T. W. Price , T. Stauch , R. Jasti , Chem. Sci. 2020, 11, 3923. 34122862 10.1039/d0sc00629g PMC8152662

[113] N. Mandal , A. Datta , Chem. Commun. 2020, 56, 15377. 10.1039/d0cc06690g 33210669

[114] R. A. Pascal Jr. , Eur. J. Org. Chem. 2004, 3763.

[115] K. Morioka , K. Wakamatsu , E. Tsurumaki , S. Toyota , Chem. Eur. J. 2022, 28, e202103694. 34762325 10.1002/chem.202103694

[116] G.‐F. Huo , T. M. Fukunaga , X. Hou , Y. Han , W. Fan , S. Wu , H. Isobe , J. Wu , Angew. Chem. Int. Ed. 2023, 62, e202218090. 10.1002/anie.202218090 36826385

[117] H. Christoph , J. Grunenberg , H. Hopf , I. Dix , P. G. Jones , M. Scholtissek , G. Maier , Chem. Eur. J. 2008, 14, 5604. 18478614 10.1002/chem.200701837

[118] S. M. Bachrach , J. Org. Chem. 2023, 88, 7962. 37294667 10.1021/acs.joc.2c02975

[119] M. Krzeszewski , H. Ito , K. Itami , J. Am. Chem. Soc. 2022, 144, 862. 34910487 10.1021/jacs.1c10807

[120] W. Fan , T. Matsuno , Y. Han , X. Wang , Q. Zhou , H. Isobe , J. Wu , J. Am. Chem. Soc. 2021, 143, 15924. 34550688 10.1021/jacs.1c08468

[121] K. Du , Y. Wang , J. Am. Chem. Soc. 2023, 145, 10763. 37092900 10.1021/jacs.3c01644

[122] Q. Zhou , X. Hou , J. Wang , Y. Ni , W. Fan , Z. Li , X. Wei , K. Li , W. Yuan , Z. Xu , M. Zhu , Y. Zhao , Z. Sun , J. Wu , Angew. Chem. Int. Ed. 2023, 62, e202302266. 10.1002/anie.202302266 37009840

[123] Y. Yu , L. Wang , C. Wang , F. Liu , H. Ling , J. Liu , Small Sci. 2023, 3, 2300040. 40213608 10.1002/smsc.202300040 PMC11935940

[124] F. Morita , Y. Kishida , Y. Sato , H. Sugiyama , M. Abekura , J. Nogami , N. Toriumi , Y. Nagashima , T. Kinoshita , G. Fukuhara , M. Uchiyama , H. Uekusa , K. Tanaka , Nat. Synth. 2024, 3, 774.

[125] S. Han , A. D. Bond , R. L. Disch , D. Holmes , J. M. Schulman , S. J. Teat , K. P. C. Vollhardt , G. D. Whitener , Angew. Chem. Int. Ed. 2002, 41, 3223. 10.1002/1521-3773(20020902)41:17<3223::AID-ANIE3223>3.0.CO;2-G 12207396

[126] S. Han , D. R. Anderson , A. D. Bond , H. V. Chu , R. L. Disch , D. Holmes , J. M. Schulman , S. J. Teat , K. P. C. Vollhardt , G. D. Whitener , Angew. Chem. Int. Ed. 2002, 41, 3227. 10.1002/1521-3773(20020902)41:17<3227::AID-ANIE3227>3.0.CO;2-T 12207397

[127] Q. Jiang , Y. Han , Y. Zou , C. Chi , J. Mater. Chem. C 2023, 11, 15160.

[128] B. Ji , Z. Qi , T. Ye , S. Li , Y. Shi , S. Cui , J. Xiao , Chem. Eur. J. 2024, 30, e202302893. 37867144 10.1002/chem.202302893

[129] Y. Chen , R. Zhou , X. Liu , C. Yang , T. Wang , F. Shi , L. Zhang , Chem. Commun. 2022, 58, 4671. 10.1039/d2cc00957a 35319555

[130] C. Maeda , T. Ema , Chem. Commun. 2025, 61, 4757. 10.1039/d4cc06307d 40035634

[131] G.‐F. Huo , W.‐T. Xu , Y. Han , J. Zhu , X. Hou , W. Fan , Y. Ni , S. Wu , H.‐B. Yang , J. Wu , Angew. Chem. Int. Ed. 2024, 63, e202403149. 10.1002/anie.202403149 38421194

[132] P. Karak , J. Choudhury , Chem. Sci. 2022, 13, 11163. 36320460 10.1039/d2sc04006a PMC9517708

[133] F. Chen , M. Melle‐Franco , A. Mateo‐Alonso , J. Org. Chem. 2022, 87, 7635. 35616330 10.1021/acs.joc.2c00129 PMC9207929

[134] F. Chen , W. Gu , A. Saeki , M. Melle‐Franco , A. Mateo‐Alonso , Org. Lett. 2020, 22, 3706. 32290661 10.1021/acs.orglett.0c01202

[135] Y. Yu , C. Wang , F.‐F. Hung , C. Chen , D. Pan , C.‐M. Che , J. Liu , J. Am. Chem. Soc. 2024, 146, 22600. 39101597 10.1021/jacs.4c06997

[136] W.‐C. Guo , W.‐L. Zhao , K.‐K. Tan , M. Li , C.‐F. Chen , Angew. Chem. Int. Ed. 2024, 63, e202401835. 10.1002/anie.202401835 38380835

[137] X. Chen , D. Tan , D.‐T. Yang , J. Mater. Chem. C 2022, 10, 13499.

[138] Y. Kondo , K. Yoshiura , S. Kitera , H. Nishi , S. Oda , H. Gotoh , Y. Sasada , M. Yanai , T. Hatakeyama , Nat. Photonics 2018, 13, 678.

[139] K. R. Naveen , H. I. Yang , J. H. Kwon , Commun. Chem. 2022, 5, 149. 36698018 10.1038/s42004-022-00766-5 PMC9814903

[140] K. R. Naveen , J. H. Oh , H. S. Lee , J. H. Kwon , Angew. Chem. Int. Ed. 2023, 62, e202306768. 10.1002/anie.202306768 37296070

[141] S. Oda , B. Kawakami , Y. Yamasaki , R. Matsumoto , M. Yoshioka , D. Fukushima , S. Nakatsuka , T. Hatakeyama , J. Am. Chem. Soc. 2022, 144, 106. 34941256 10.1021/jacs.1c11659

[142] J. Full , S. P. Panchal , J. Götz , A.‐M. Krause , A. Nowak‐Król , Angew. Chem. Int. Ed. 2021, 60, 4350. 10.1002/anie.202014138 PMC7898935 33244880

[143] Y. Yu , L. Wang , D. Lin , S. Rana , K. S. Mali , H. Ling , L. Xie , S. De Feyter , J. Liu , Angew. Chem. Int. Ed. 2023, 62, e202303335. 10.1002/anie.202303335 36964955

[144] X. Zeng , X. Luo , G. Meng , X. Wang , D. Zhang , L. Duan , Angew. Chem. Int. Ed. 2025, 64, e202423670. 10.1002/anie.202423670 39780447

[145] Z. Zhang , W. Hu , Z. Liu , Y. Tsutsui , Y. Murata , S. Seki , T. Hirose , J. Am. Chem. Soc. 2025, 147, 25978. 40633960 10.1021/jacs.5c08688

[146] Y. Zhu , C. Lin , C. Zhao , Y. Ou , Z. Li , K. Zhu , J. Wang , Angew. Chem. Int. Ed. 2024, 64, e202420073. 10.1002/anie.202420073 39545719

[147] K. Y. Chernichenko , V. V. Sumerin , R. V. Shpanchenko , E. S. Balenkova , V. G. Nenajdenko , Angew. Chem. Int. Ed. 2006, 45, 7367. 10.1002/anie.200602190 17001717

[148] E. Licandro , S. Cauteruccio , D. Dova , Adv. Heterocycl. Chem. 2016, 118, 1.

[149] C.‐B. Huang , A. Ciesielski , P. Samorì , Angew. Chem. Int. Ed. 2020, 59, 7319. 10.1002/anie.201914931 31898855

